# Effects of process intensification on homogeneity of an IgG1:κ monoclonal antibody during perfusion culture

**DOI:** 10.1007/s00253-024-13110-9

**Published:** 2024-03-26

**Authors:** George Liang, Chikkathur N. Madhavarao, Caitlin Morris, Thomas O’Connor, Muhammad Ashraf, Seongkyu Yoon

**Affiliations:** 1 Division of Product Quality Research, OTR/OPQ, CDER/FDA, Silver Spring, MD USA; 2 Department of Chemical Engineering, University of Massachusetts Lowell, Lowell, MA USA

**Keywords:** CHO-K1 cell line, VRC01 mAb, Micro heterogeneity, N-glycosylation, Protein integrity, Isoelectric point

## Abstract

**Abstract:**

The pharmaceutical industry employs various strategies to improve cell productivity. These strategies include process intensification, culture media improvement, clonal selection, media supplementation and genetic engineering of cells. However, improved cell productivity has inherent risk of impacting product quality attributes (PQA). PQAs may affect the products’ efficacy via stability, bioavailability, or in vivo bioactivity. Variations in manufacturing process may introduce heterogeneity in the products by altering the type and extent of N-glycosylation, which is a PQA of therapeutic proteins. We investigated the effect of different cell densities representing increasing process intensification in a perfusion cell culture on the production of an IgG1-κ monoclonal antibody from a CHO-K1 cell line. This antibody is glycosylated both on light chain and heavy chain. Our results showed that the contents of glycosylation of IgG1-κ mAb increased in G0F and fucosylated type glycans as a group, whereas sialylated type glycans decreased, for the mAb whole protein. Overall, significant differences were observed in amounts of G0F, G1F, G0, G2FS1, and G2FS2 type glycans across all process intensification levels. G2FS2 and G2 type N-glycans were predominantly quantifiable from light chain rather than heavy chain. It may be concluded that there is a potential impact to product quality attributes of therapeutic proteins during process intensification via perfusion cell culture that needs to be assessed. Since during perfusion cell culture the product is collected throughout the duration of the process, lot allocation needs careful attention to process parameters, as PQAs are affected by the critical process parameters (CPPs).

**Key points:**

*• Molecular integrity may suffer with increasing process intensity.*

*• Galactosylated and sialylated N-glycans may decrease.*

*• Perfusion culture appears to maintain protein charge structure.*

**Supplementary Information:**

The online version contains supplementary material available at 10.1007/s00253-024-13110-9.

## Introduction

Therapeutic proteins such as enzymes (Fratz-Berilla et al. 2017; Hennigan and Lynch 2022), hormones (Colditz 1998), and monoclonal antibodies (mAbs) form important classes of biologic drugs used for the treatment of human diseases. Traditionally, these therapeutic proteins are produced recombinantly using mammalian cells, most commonly from Chinese hamster ovary (CHO) cells. These cells are grown in bioreactors, in either batch or fed-batch cultures (O'Flaherty et al. 2020) or perfusion cultures (Santos et al. 2019). During production, these large protein molecules or biologic drugs, also undergo certain modifications that may be critical to their mechanism of action in vivo. These modifications are considered critical quality attributes (CQAs), and one such CQA is the glycosylation, i.e., the addition of carbohydrate chains on specific amino acid residues such as asparagine (N-linked) or serine/threonine (O-linked) of the proteins. N-linked glycosylation in mAbs affects the pharmacological properties such as stability, in vivo bioactivity, bioavailability and efficacy (Beck and Liu 2019; Higel et al. 2019). Notably, variations in N-linked glycans may occur during production due to variations in the carbohydrate chains (or glycans) (Fisher et al. 2019). This heterogeneity in N-glycans usually occurs between batches due to variations in process parameters, with changes in raw materials used in the cell culture or alterations in the genes of the CHO cell line. Maintaining the product homogeneity is necessary for a mAb to ensure drug safety and efficacy. Two kinds of glycan heterogeneity have been reported, namely (1) micro heterogeneity and (2) macro heterogeneity (Čaval et al. 2021). Micro-heterogeneity deals with different types of glycans that occur at the same asparagine residues due to either chemically induced reactions or enzymatic reactions (Edwards et al. 2022; Kunert and Reinhart 2016). Micro-heterogeneity has been associated with the safety, efficacy, and pharmacokinetics and pharmacodynamics (Sissolak et al. 2019). By contrast, macro-heterogeneity of glyco-proteins may be the result of variation in site-specific glycosylation, i.e., the transfer of the oligosaccharides to specific amino acid residues on the protein (Čaval et al. 2021; Planinc et al. 2016). For these reasons, monitoring the glycan composition and structure during mAb production can be useful for mAb product quality.

For increasing cell productivity, batch processes have been extensively investigated while maintaining certain N-linked glycan critical quality attributes (Ehret et al. 2019; Fan et al. 2015; Mellahi et al. 2019; Monteil et al. 2016; Parhiz et al. 2019; Yuan et al. 2019). For maintaining homogeneity and controlling the extent of specific glycan types, various supplements have been used (e.g., kifunensine, 2-F-peracetyl fucose, galactose, manganese or uridine) (Ehret et al. 2019). Similarly, different amino acids (leucine, arginine) and glucose balance were used to control N-linked glycan profiles (Fan et al. 2015). In addition, process conditions such as temperature shifts (Mellahi et al. 2019), differences in agitation speeds and pH of the media (Parhiz et al. 2019), and bioreactor types such as orbitally shaken bioreactors (OSRS) versus stirred tank bioreactors (STRs) have also been studied to understand the impact on N-linked glycosylation (Monteil et al. 2016). Genetic manipulation of the cells to the extent of knocking out specific glycosylation-related genes have also been conducted to control glycan heterogeneity (Yuan et al. 2019).

Importantly, byproduct formation (e.g., lactate and ammonia) in batch and fed-batch processes is known to impact product quality and protein production (MacDonald et al. 2022; O'Flaherty et al. 2020). To minimize the build-up of these byproducts and decrease their impact on cell culture performance, batch production has been replaced by perfusion cultures which produce more homogenous product (O'Flaherty et al. 2020). Moreover, perfusion cultures are preferable due to their high volumetric output and shorter exposure time of the product to the production environment, which in turn leads to minimal risk of product degradation or alteration (Huang et al. 2010). Perfusion culture apparatuses facilitate continuous production of proteins that provides better speed-to-market product, decreased cost and flexible manufacturing (Chen et al. 2018; Rathore et al. 2022). In a continuous upstream manufacturing process, the key considerations are typically (1) cell-specific productivity (qP), (2) viable cell density (VCD), and (3) time span of the bioreactor ‘idle’ stage (Chen et al. 2018). A bridge between fed-batch and continuous manufacturing has also been attempted via a perfusion system coupled to a hollow fiber in tangential flow filtration (TFF) mode (Stepper et al. 2020). Pre-staging the process from the perfusion reactor harvest (N-1 perfusion bioreactor) did not show any variation in product quality of a mAb while targeting a specific product quality profile (Stepper et al. 2020). In a targeted feeding approach using a perfusion culture to influence glycosylation, target cell specific consumption rate, and cell specific perfusion rate (CSPR) in CHO cells resulted in different glycan profiles with different feeding regimes, even though a specific viable cell density (VCD) was used (Zhang et al. 2021). Bolus feeding during pre-induction period of the batch culture and harvesting earlier in the perfusion culture to increase specific N-linked glycosylation profiles has shown an inherent tradeoff between antibody titer and N-linked glycan profile (Mellahi et al. 2019). Thus, even for perfusion cell culture processes, under high cell density conditions, there is a potential risk to product quality and homogeneity. The previous studies characterized N-linked glycan profiles at a single VCD (either at the end of the culture or at a certain VCD) that did not provide information on how higher cell density during perfusion culture could impact protein N-glycosylation homogeneity and overall mAb molecular integrity. For these reasons, the effect of process intensification was investigated to produce a broadly neutralizing IgG1-κ monoclonal antibody (VRC01) against HIV from a CHO-K1 cell line and variation in cell density was carried out in a perfusion cell culture to understand the effect of higher cell densities on product homogeneity.

## Materials and methods

### Materials

#### Reagents

All chemicals and reagents used in this study were of analytical grade and stored at the recommended storage conditions during our investigations. Sodium bicarbonate, glucose, sodium phosphate, sodium citrate, sodium chloride, dithiothreitol (DTT), maltose, acetonitrile, Tris–HCl, glycine, sodium dodecyl sulfate (SDS), 2-aminobenzamide (2-AB), Borane-2-methylpyridine complex 95%, urea, dimethyl sulfoxide (DMSO), acetic acid and ammonium formate were purchased from Sigma-Aldrich (St. Louis, MO, USA), and stored at room temperature except DTT (stored at − 20 °C) and borane-2-methylpyrimidine complex 95% (stored at 4 °C). Organic solvents such as ethanol, methanol, and acetonitrile were of HPLC grade, purchased from Millipore Sigma (Burlington, MA, USA) and stored at room temperature in fire-proof cabinets. Nuclease free water, genomic DNA purification kit and L-glutamine were purchased from Thermo Fisher Scientific (Waltham, MA, USA). Nuclease free water was stored at room temperature and glutamine at − 20 °C. RNase A and proteinase K from the DNA purification kit were stored at − 20 °C, and the cell lysis, elution, washing, and binding buffers were stored at room temperature. N-glycan sample preparation kit and 2x Luna Universal qPCR Master Mix were purchased from New England Biolabs (Ipswich, MA, USA). The master mix was stored at − 20 °C. Reagents from within the N-glycan sample preparation kit such as 10% NP-40, 10X denaturation buffer and 10X reaction buffer were stored at − 80 °C, and the PNGaseF enzyme at 4 °C. APTS (8-Aminopyrene-1,3,6-Trisulfonic Acid) labeling kit and peptide markers of pI 10, 9.5, 7, 5.5, and 4.1 were purchased from AB Sciex (Redwood City, CA, USA). APTS kit was stored at 4 °C and peptide markers at − 20 °C. Agencourt CleanSEQ magnetic beads were purchased from Beckman Coulter (Brea, CA, USA) and stored at 4 °C. Deionized and filtered Milli-Q water of 18.4 MΩ resistance from a Milli-Q purification system (EMD Millipore, Burlington, MA, USA) was used in the preparation of all aqueous buffers and reagents. APTS labeled N-glycan standards: G2FS2, Man 5, G0F, Man 9, G1F, Man 7, G2, Man 8, G2F, G0, G2FS1, G1, and 2-AB labeled N-glycans standards: G2FS2, Man 5, G0F, Man 9, G1F, Man 7, G2, Man 8, Man 6, G2F, G0, G2FS1, and G1 were purchased from Agilent Technologies (Santa Clara, CA, USA) and all glycan standards were stored at − 20 °C. Laemmli protein denaturation and loading buffer (2X) were purchased from Bio-rad (Hercules, CA, USA) and stored at 4 °C. Primer of sequence CCG ACT CGA GNN NNN NAT GTG G was purchased from IDT Technologies (Burlington, MA, USA) and stored at − 20 °C.

#### Cell line

A mammalian cell line of Chinese hamster ovary origin, CHO-K1, capable of producing a broadly neutralizing recombinant human monoclonal antibody against Human Immunodeficiency Virus (HIV), was developed by the Vaccine Research Center, NIAID, of the National Institutes of Health, Bethesda, MD, USA, and named VRC01. This cell line was shared with FDA for regulatory science and research under a material transfer agreement. In our studies, we have used this CHO-K1 cell line that produces an IgG1-κ monoclonal antibody known to broadly neutralize HIV strains (Li et al. 2011; Su et al. 2014).

### Production of mAbs under different process intensities

#### Mammalian cell culture

A single vial from a working cell bank of CHO-K1 (VRC01, NIH) cell line was thawed and expanded in shake flask cultures in an incubator set to 37 °C that has 5% CO_2_ mixed air circulation. ActiPRO™ medium (Cytiva, Marlborough, MA, USA) was reconstituted as per manufacturer’s recommendation with sodium bicarbonate salt for buffer and 6 mM L-glutamine (Invitrogen, Carlsbad, CA, USA), adjusted to pH 7.05, then filter sterilized for cell culture and for production of the mAb. After the cells were expanded to ≥ 10E09, the cells were inoculated [(0.5 ± 0.15) × 10E06 · mL^−1^] to a reusable 5L glass vessel (~ 2L working volume) bioreactor equipped with stainless steel headplate and supports, operated with a DASGIP controller system (Eppendorf, Hamburg, Germany). The bioreactor run was aided with a perfusion system made up of Repligen ATF2 device and C24 rate controller (Repligen, Waltham, MA, USA). The ATF2 utilized a hollow fiber filter of 0.2 µm pore size and 0.1 m^2^ filter area. The bioreactor was equipped with an electric heat jacket and an in-line temperature probe to maintain temperature and set to operate at 37 °C, pH of 7.0 ± 0.1 and dissolved oxygen (DO) content of 30% of air saturation. An on-line pH probe (Mettler Toledo, Columbus, OH, USA) and a DO probe (Hamilton, Reno, NV, USA) were used to monitor and maintain pH and DO. The pH of medium in the reactor was maintained with a combination of sparged CO_2_ gas and 7.5% sodium bicarbonate buffer (Sigma Aldrich, St. Louis, MO, USA). The DO content was maintained by sparging oxygen and air, and, by stirring the tank with a pitched-blade impeller operated at 120 rpm. Perfusion was started on day 3 of the culture at the rate of half working vessel volume (1 L) per day. The perfusion rate was increased incrementally throughout the culture duration, and these rates are shown in Table 1. Perfusates were collected for analysis corresponding to three different process intensities such that the viable CHO cell densities were 15 ± 1, 20 ± 1 and 25 ± 1 × 10E06 cells/mL and finally the run was terminated after harvesting at 26 ± 1 × 10E06/mL cells.

**Table 1:** Perfusion Schedule and the viable cell density (VCD) of the bioreactor corresponding to the samples analyzed

Day number^a^	Perfusion rate (Vessel volume/day; VVD)	VCD of samples analyzed,^b^(10E06/mL)
3	0.5	
4	1.0	
5	1.0	
6	1.5	
7	1.5	
8	2.0	14.43
9	2.0	15.66
10	3.0	20.23
11	3.0	20.47
12	4.0	
13	3.0	
14	4.0	24.00
15	4.0	26.00

^a^Perfusion started on day 3; ^b^VCD given is the average of NOVA Flex 2 measured and the capacitance (Aber biosensor) probe determined value

### Antibody purification

Perfusates corresponding to different cell densities of bioreactor operation were first purified through a protein A column (Cytiva, Marlborough, MA, USA) to capture the mAb and to remove host cell proteins (HCP). A further cleaning up of the captured mAb was performed on an anion exchange resin (Diethylaminoethyl sepharose, DEAE) column of 5 mL size (Cytiva, Marlborough, MA, USA) to remove nucleic acid contamination, which gave a mAb purity ≥ 98%. For protein A capture, 20 mM sodium phosphate buffer pH 7.0 was used as the binding buffer, and 200 mM sodium citrate pH 3.0 as the elution buffer. For the DEAE column, 5 mM sodium phosphate buffer pH 7.0 was used as the binding buffer and 5 mM sodium phosphate buffer pH 7.0 containing 200 mM sodium chloride was used as elution buffer. Only one peak corresponding to the mAb was eluted during the gradient elution from the DEAE column (from 0 to 200 mM NaCl). Most of the mAb eluted by 75% of the gradient (≤ 150 mM NaCl) and no other peak was detected at 100% of elution buffer ran for another 8 column volumes (Figure S1). After each purification, an SDS-PAGE (sodium dodecyl sulfate–polyacrylamide gel electrophoresis) analysis was performed to ascertain the purity of mAb (Figure S2A). Additionally, we performed capillary electrophoresis with SDS (CE-SDS) analysis to determine the purity of the mAb protein after DEAE column purification.

#### Purification of light chain (LC) and heavy chain (HC) polypeptides from the mAb

First, we developed a size exclusion chromatography (SEC) method to separate and distinguish the LC and HC polypeptides of the VRC01 mAb. Purified mAb protein (1 mg) was buffer exchanged (twice, each time diluting tenfold v/v, in the 1x denaturing buffer) using Amicon 10 kDa filters (Millipore Sigma, St. Louis, MO, USA). The 1x denaturing buffer consisted of 2% SDS, 50 mM Tris–HCl, 1 mM DTT, 50 mM NaCl, pH 9.0. Then excess dithiothreitol (DTT) was added to make up to 2-, 5- and 10-mM concentration of DTT in 3 samples of analysis, respectively. These samples were incubated for 15 min on an Eppendorf heating block (50 °C, 400 rpm) cooled to room temperature and SEC was performed using a Superdex®200 Increase HiScale® 26/40 column on an AKTA avant 25 chromatography system fitted with a 5 mL super loop (Cytiva, Westborough, MA, USA). The column was pre-washed with Milli-Q water (18.2 MΩ), followed by pre-equilibration (1 CV) with running buffer consisted of 0.05% SDS, 50 mM Tris–HCl, 1 mM DTT, 50 mM NaCl, pH 9.0. Elution was performed at a flow rate of 3.0 mL/min for 212 mL and 2 mL fractions were collected into a deep well 96-well plate (at 6 °C). The fractions corresponding to the three distinct peaks (Figure S3) were concentrated separately using Amicon 10 kDa centrifugation filters (Millipore-Sigma). Protein concentrations were determined using Pierce™ BCA Assay (Thermo Scientific, Waltham, MA, USA) on a BioTek Synergy H1 plate reader (Agilent Technologies, Santa Clara, CA, USA). SDS-PAGE analysis was performed on the polypeptides belonging to the three peaks of SEC to ascertain the isolation of LC (~ 25 kD) and HC (~ 50 kD) polypeptides and the peak that contained the partially reduced and separated mAb protein (~ 75kD) (Figure S4). Based on the resolution, purification and the recovery (Figure S4 and Table S1), treatment with 5 mM DTT was adopted for isolating the LC and HC peptides of all the mAb samples of the current study. The isolated LC and HC polypeptides of the mAb samples were cold stored (− 20 °C) until further analysis of N-glycans by HPLC as described in the following sections.

### Verification of DNA contamination in mAb by qPCR

The contamination level of the host cell nucleic acid content in the purified mAb was determined by qPCR analysis. The assay was based on a previous work (Kang et al. 2011). Briefly, a standard curve from 5 nanogram to 500 attogram of DNA was generated from a CHO-K1 cell DNA stock (IDT, Coralville, Iowa, USA). The stock DNA was produced from host cells and purified using a genomic DNA purification commercial kit (Thermo Scientific, Waltham, MA, USA). Then, real-time qPCR detection and quantification of target DNA sequences were performed on the SYBR®/FAM channel on an Applied Biosystems qPCR instrument (Thermo Scientific) using the Luna Universal qPCR Master Mix (NEB, Ipswich, MA, USA) (Figure S5).

### Verification of mAb purity and Integrity by CE analysis

#### CE-SDS analysis

All CE analyses were performed on PA800 Pharmaceutical Analysis Capillary Electrophoresis system (AB Sciex, Redwood City, CA, USA). CE analysis was conducted under reducing and non-reducing conditions as previously reported (Fratz-Berilla et al. 2017; Parhiz et al. 2019). Under reducing conditions, another comparative mAb (a biosimilar to Humira or Adalimumab obtained from the University of Massachusetts – Lowell) was also run to confirm the light chain glycosylation on the VRC01 mAb. CE-SDS under non-reducing conditions was performed to determine the mAb integrity.

#### cIEF analysis of charge variants and pI determination

We have performed capillary iso-electric focusing (cIEF) analysis as previously described (Parhiz et al. 2019) using a neutral capillary to determine the isoelectric point (pI) and charge variants. Briefly, first, cIEF assay conditions were standardized by performing sample preparation in 1M, 2M, and 3M urea containing cIEF gel. Based on the resolution and the sharpness of the peaks, 3M urea-cIEF gel was chosen for the analysis (Figure S6). Electro-focusing was performed using broad range ampholytes (pH 3–10; Pharmalyte™ carrier ampholytes by Cytiva). All charge variant peaks migrated within the marker pI of 9.5 and 7.0. Therefore, all samples were analyzed with the marker peptides of pI 9.5 and 7.0. Peaks were manually identified on the electropherograms within the two flanking pI markers (9.5 and 7.0). The migration time and corresponding peak area (%) values were obtained for all peaks (including the flanking pI markers) using 32 Karat software (AB Sciex). Migration times of each charge variant peak was converted into pI units using the migration time difference between the two flanking pI markers. The determined pI values were verified against a standard curve to agree within a standard deviation of ≤ 0.14 pI units among all the peaks analyzed. The standard curve was generated using peptide markers of pI 10, 9.5, 7, 5.5 and 4.1 (*n* = 20) (Figure S6).

### Analysis of microheterogeneity of mAb produced under different process intensities

#### N-linked glycan isolation

The N-glycans were isolated using a kit supplied by New England Biolabs (NEB, Ipswich, MA, USA). Samples containing 20 µg mAb were first denatured using 1x denaturation buffer for 10 min at 100 °C. Then cooled on ice for 1 min followed by release of the glycans by treating with PNGaseF enzyme. First, conditions for separation of glycans from the mAb were investigated by varying the amount of PNGaseF required and varying the treatment time. The mAb treatment with PNGaseF for 1 h or 2 h (Figure S7) was performed at 37 °C. As per the manufacturer-recommended protocol, we used the 1 × reaction buffer and 1% NP-40 in the de-glycosylation procedure. Then, SDS-PAGE analysis was performed to ensure that a downward shift in the mAb bands was observed to the expected molecular weight, indicative of complete deglycosylation.

#### Labeling N-glycans with APTS and CE analysis

N-glycans isolated from the mAb samples (250 µg protein/sample) were labeled with APTS (8-Aminopyrene-1,3,6-Trisulfonic Acid) using a kit supplied by AB Sciex (Redwood City, CA, USA) as per the manufacturer’s protocol. Maltose was used as an internal standard in all samples. For the dye and n-glycan reaction, the samples were incubated overnight at room temperature to decrease de-sialylation effect of higher temperatures. Following APTS labeling, the N-glycan samples were removed off excess dye using Agencourt CleanSEQ magnetic beads (Beckman Coulter, Brea, CA, USA) and 87.5% acetonitrile solvent. A magnetic rack for microcentrifuge tubes facilitated retention of beads in the Eppendorf vials for easy removal of solvents. First, the beads were treated with the solvent and the solvent was removed. Then, the samples were mixed with the beads and 87.5% acetonitrile solvent was added (at a volume ratio of 1: ≥ 7, sample: solvent) and mixed with the beads containing glycan sample. Placing the vials on the magnetic rack facilitated retention of beads and easy removal of solvent. Beads with glycan samples were washed two times with 87.5% acetonitrile solvent. Glycans were eluted from the beads in 20 µL of Milli-Q water. This process removed excess dye and gave better glycan profiles. APTS labeled N-glycan standards (Agilent Technologies, Santa Clara, CA, USA) were used to identify individual peaks (Figure S8). Glycans were analyzed as per the manufacturer’s recommendation using an nCHO capillary and glycan analysis kit (AB Sciex, Redwood City, CA, USA). Glycans were detected and quantified using a 488-nm laser induced fluorescence (LIF) detector on a PA800 Capillary Electrophoresis system (AB Sciex, Redwood City, CA, USA).

#### Labeling N-glycans with 2-AB and cleaning

The N-glycan samples isolated from the whole mAb samples as well as the LC and HC polypeptides were derivatized with 0.35 M 2-AB (Sigma Aldrich, St. Louis, MO, USA) and 1M Borane-2-methylpyridine complex 95% (Sigma Aldrich, St. Louis, MO, USA) dissolved in a mixture of 70% DMSO (Sigma Aldrich, Burlington, MA, USA) and 30% Acetic acid (Sigma Aldrich, Burlington, MA, USA), for 2.5 h at 65 °C. The labeled N-glycan samples were then cleaned to remove excess dye by using Agencourt CleanSEQ magnetic beads (Beckman Coulter, Brea, CA, USA). Briefly, 200 µL of thoroughly mixed magnetic beads were taken in 1.5 mL Eppendorf microcentrifuge tubes and bead separations were performed in a Permagen Labware (Permagen, Peabody, MA, USA) equipped with magnets to facilitate separation of beads from a solution. First, the magnetic beads were prepared as follows: 200 µL of thoroughly mixed magnetic beads were taken in microcentrifuge tubes and placed in the Permagen labware for 3 min, then the solvent was removed. The beads were then washed with HPLC grade acetonitrile (100%), 3 times with 3-min incubation intervals. The 2-AB labeled samples were diluted ninefold with 100% acetonitrile, mixed thoroughly with prepared magnetic beads and placed in the Permagen labware for 3 min. After removing the supernatants, the beads were washed with 96% acetonitrile for 3 times with 3-min incubation intervals. The beads were then finally washed with Milli-Q water to elute the 2-AB labeled N-glycans from the beads. The purified labeled N-glycans were dried for 2 h in a Speed Vac freeze-dryer (Thermo Scientific, Waltham, MA, USA) and resuspended in 15 µL of Milli-Q water and directly injected into an HPLC column.

#### N-linked glycan characterization by HPLC analysis

Analysis of cleaned 2-AB labeled N-glycan samples was performed using a previously published method (Sha et al. 2020) with a slight modification to accommodate more complex N-glycan profiles. This method involves a buffer A of pH 4.5 made up of 100 mM ammonium formate (Millipore Sigma, Burlington, MA, USA), and a buffer B of 100% acetonitrile (Millipore Sigma, Burlington, MA, USA). Separation of N-glycans was performed on a Glycan BEH Amide 150 mm column-130 A with 1.7 µm beads and dimensions of 2.1 mm × 150 mm (Waters, Milford, MA, USA). A guard column was also used (Acquity UPLC Glycan BEH Amide Vanguard column; Waters, Milford, MA, USA). The column was pre-equilibrated with 25% of buffer A at 60 °C column temperature. About 1 µL of the N-glycan sample was injected and elution was performed with the following conditions: (a) for 0–62.50 min, buffer A was varied from 25 to 40% at a flow rate of 0.3 mL/min; (b) for the next 7.5 min, the flow rate was reduced to 0.1 mL/min and the gradient increased from 40 to 100% of buffer A; (c) for the next 6 min, 100% buffer A was run to remove any strongly bound glycans; (d) the flow rate was then ramped up over 10 min from 0.1 to 0.2 mL/min while decreasing the percent of buffer A from 100 to 25%.

The column was pre-equilibrated to the starting condition, by gradually increasing the flow rate from 0.2 to 0.3 mL/min in 5 min and keeping the 25% of buffer A running for an additional 30 min before the next sample injection. After every 9 samples, a blank consisting of 25% ammonium formate and 75% acetonitrile was injected to ensure there was no sample carryover between injections. A standard mix consisting of 13 types of 2-AB labeled N-glycan standards was used and separated by the same HPLC procedure to identify the N-glycan peaks (Figure S9): G2FS2, Man 5, G0F, Man 9, G1F (G1F’), Man 7, G2, Man 8, Man 6, G2F, G0, G2FS1, and G1 (G1’). All the standards were purchased from Agilent Technologies (Santa, Clara, CA, USA). HPLC analysis was performed on an Agilent 1260 high pressure liquid chromatography system (Agilent, Santa Clara, CA, USA) consisting of Agilent 1260 UV detector (catalog no G4212B, serial no. DEAA301559), a fluorescence detector (catalog no.G1321B, serial no. DEABW04335), an autosampler (catalog no. G1329B, serial no. DEAB306274), pumps (catalog no. G1312B, serial no. DEABM02021), a degasser (catalog no. G1379B, serial no. JP02415768), a column thermostat (catalog no. G4212B, serial no. DEAA301559) and a chiller (catalog no. G1330B, serial no. DEBAK33683).

### Statistical analyses

Quantitative differences in the composition of N-glycan types were evaluated by analysis of variance (ANOVA) of the integrated peak area (%) values normalized to total peak area of N-glycan types. The percent peak areas were determined for every glycan elution profile of CE analysis, using 32 Karat software (AB Sciex). Then, ANOVA was performed on peak area (%) values of the samples (*n* = 3 to 9) corresponding to each of the process intensity levels followed by comparison of means by Tukey–Kramer HSD. The relationship between N-glycan content and the viable cell density for different types of N-glycans was evaluated by regression analysis of variance (RANOVA) to ascertain the risk to the model and the risk to consider the slope ≠ 0. For these statistical analyses, we used the software JMP, version 16.0.0 (Cary, NC, USA).

Principal component analysis (PCA) on the data was performed to ascertain the association of process variables and the measured quality attributes of the mAb. The analysis with 3 components explained > 90% of the variability in the data. The relative distance between the parameters on the two component plots from the PCA analysis was used to generate a hierarchical clustering analysis (HCA) by a single linkage method. Thereafter, HCA clusters these parameters as groups to understand which parameters are closely related and which are distantly related. We performed clustering independently for relative distance of 0.01, 0.02, 0.03, 0.04, and 0.05 between the parameters. When the parameters were separated by shorter (0.01–0.03) relative distance, too many groups were observed. Conversely, when the parameters were separated by longer (> 0.05) relative distance, there was only one group, not distinguishing any parameters. Therefore, the parameters were clustered based on the relative distance of ≤ 0.05, which gave 3 distinct groups of parameters. We used a multivariate data analysis (MVDA) tool, SIMCA (Version 17, Sartorius, Cambridge, MA, USA) for these analyses.

## Results

### Molecular purity of the VRC01 mAb

Perfusates of the cell culture process held at different viable cell densities, such as (15 ± 1) × 10E06 cells/mL, (20 ± 1) × 10E06 cells/mL, (25 ± 1) × 10E06 cells/mL and the cell bleeds obtained at 15 × 10E06 cells/mL and 20 × 10E06 cells/mL were collected and the resultant mAbs purified by two-step chromatography — protein A capture followed by DEAE anion exchange polishing. First, we investigated the purity and integrity of the mAb by CE-SDS under reducing and non-reducing conditions, respectively. Host cell DNA contamination evaluated by qPCR showed that purification by 2-step chromatography removed the DNA contamination to less than detectable quantities by qPCR (≤ 0.5 picogram/mg protein).

**Fig. 1:**
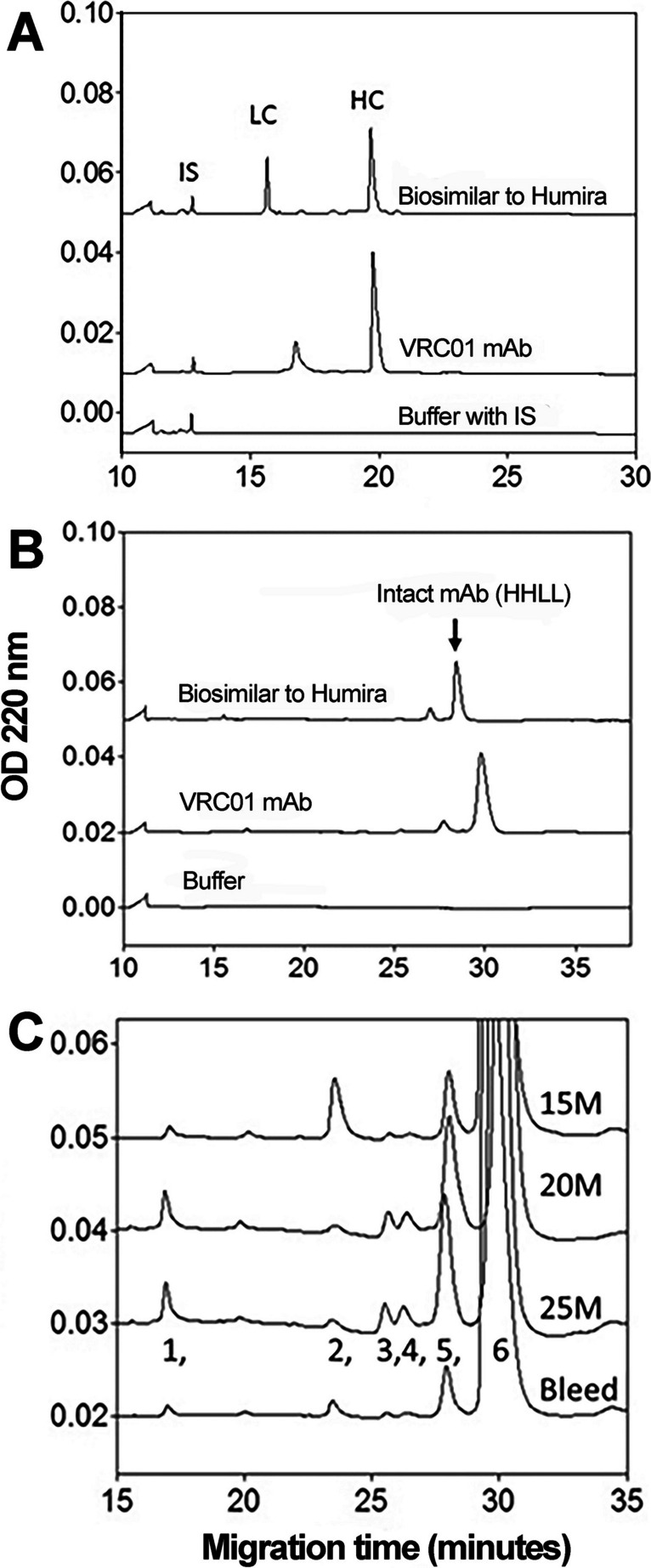
CE-SDS analysis of the mAb under reducing (**A**) and non-reducing (**B** and **C**) conditions. Comparison of VRC01 mAb with a biosimilar to Adalimumab^TM^ (Humira^TM^) shows slower migration of light chain (LC) of VRC01 that has a relatively broader peak indicative of glycosylation (**A**). Heavy chain (HC) does not show any noticeable difference between the two mAbs; internal standard (IS; 10 kD polypeptide) shows comparable migration time in samples with or without the mAb. Even under non-reducing conditions the intact VRC01 mAb molecule (consisting of 2H and 2L chain polypeptides) migrates slower than biosimilar to Humira (**B**). VRC01 mAb molecule purified from the bioreactor broth under different viable cell densities (15–25 × 10E06 cells/mL and cell bleed), are compared for the molecular integrity, and the expanded view of the stacked profiles are shown in (**C**). Molecular integrity analysis of these results is presented in Table 2

Both SDS-PAGE and CE-SDS performed under reducing conditions showed > 98% purity of the VRC01 mAb. Furthermore, CE-SDS performed under reducing conditions showed a much broader peak width (i.e., more heterogeneity) for the glycosylation of the light chain than the non-glycosylated light chain of an IgG of a biosimilar to adalimumab. While aggregation of polypeptides can produce broader peaks, considering that the mAb was highly purified, any aggregation of light chain (LC) polypeptides can produce peaks that migrate closer to heavy chain (HC) or beyond, which was not evident by the CE-SDS profiles under reducing conditions. This broadness of the peak may correspond to the degree of glycosylation and composition of the N-glycans of the light chain as one of the probabilities. This variation in the broadness seen across the samples from different process intensity levels and slower migration of the light chain of the IgG1-κ monoclonal antibody (VRC01) was evident in comparison with a biosimilar of adalimumab (Fig. 1A). The broadness of the LC appears to decrease at a cell density of 20 × 10E06 cells/mL when compared to 15 × 10E06 cells/mL and quantitative analysis of the LC peaks showed statistically significant difference for the peak width at 50% height (Figure S2B and C). The heavy chain migration data was found quantitatively comparable across different cell densities for peak width at 50% height (Figure S2C). However, CE-SDS performed under non-reducing conditions showed that the mAb appeared to lose some structural integrity as several minor peaks were detectable besides the major peak attributable to an intact mAb consisting of 2 HC and 2 LC (HHLL).

### Molecular integrity of the VRC01 mAb

Figure 1B shows electropherograms of the CE-SDS analysis performed under non-reducing conditions for VRC01 mAb with its comparator (a biosimilar to Humira™, Adalimumab™). The detectable peaks of VRC01 migrate slower than that of its comparator under non-reducing conditions also, indicating higher glycosylation, despite both belong to IgG1. Purified VRC01 mAb produced under different viable cell densities was analyzed by CE-SDS under non-reducing conditions. An expanded view of the stacks of VRC01 electropherograms is given in Fig. 1C that depicts the quantitative variations in the minor peaks, along with a major peak (as seen in Fig. 1B). The electropherogram shows several minor peaks and a major peak between 15 and 35 min of migration time. The major peak that elutes at the end may be attributed to an intact mAb (HHLL). These peaks are numbered 1 through 6 for comparative purposes (Fig. 1C). Based on the molecular association of the H and L chains, these minor peaks may be attributed to individual or combinations of H and L chains (such as L, H, HL, HH, HHL, and the intact HHLL). However, we label them as peaks 1 through 6 since we do not have direct evidence for such molecular association of H and L chain migration behaviors in CE-SDS. We determined the percent integrity of the mAb by quantifying the area under the curve of all contributing peaks from the CE-SDS electropherogram normalized to percent area for every sample (Cao et al. 2021; Dada et al. 2017; Salas-Solano et al. 2006).

The results in Table 2 show variability in the molecular integrity (intact molecule consisting of 2 HC and 2 LC polypeptides) of VRC01 mAb seen across different cell densities and in the mAb purified from the cell bleed. These results show that molecular integrity of mAb appeared to decrease as measured by peak area (%) at higher process intensification. The peak area (%) of the largest peak (corresponding to 2HCs and 2LCs) was lower for mAb samples of higher cell densities of 20 and 25 × 10E06 cells/mL compared to the 15 × 10E06 cells/mL values. However, cell bleeds exhibited a higher integrity of overall ~ 93%. Approximately 89% of intact mAb was found at cell density of 15 × 10E06 cells/mL, that is ~ 10% higher than the cell densities of 20 and 25 × 10E06 cells/mL. A comparison of peak area (%) for peaks 1, 5, and 6 of the purified mAb from all processes was carried out by ANOVA followed by comparison of means, which showed significant differences (*p* < 0.005). The mean peak area (%) comparison by Dunnett’s HSD of purified mAb for cell densities of 20 and 25 × 10E06 cells/mL was significantly different (*p* < 0.01) than that of cell bleed samples while the peak area (%) of 15 × 10E06 cells/mL process was not significantly different. In addition, the viability of cells was observed to plateau from ~ 95% to between 85 and 90% at higher cell densities (Figure S10). A concomitant steady increase in lactate by-product was also observed in the early phase, which further increased at higher intensification levels, even though the cell culture was performed under perfusion conditions (Figure S11F). The decrease in cell viability at higher cell densities may be due to the increase in lactate byproduct that may have caused cell apoptosis (Fu et al. 2016). The concentrations of major metal ions (Na, K and Ca) did not deviate significantly throughout the run although the osmolality showed deviations (between 440 and 520 mOsm/kg) through the run. The deviation in osmolality may be attributable to the variation in glucose, lactate, glutamine, glutamate, and ammonium concentrations (Figure S11 and 12). However, variations in the added sodium bicarbonate buffer including those solutes which were not considered for calculation (from a proprietary chemically defined medium, which typically consists of 50–100 different ingredients) could also contribute to the variations in osmolality.

**Table 2:** Molecular Integrity of VRC01 mAb determined by CE-SDS under non-reducing conditions (*n* ≥ 4)

Detected peak number^b^	Process intensification level as VCD (10^6^ · mL^−1^)								
	15 ± 1		20 ± 1		25 ± 1			Cell bleed^a^	
	Migration time (min)	Peak area %	Migration time (min)	Peak area %	Migration time (min)	Peak area %		Migration time (min)	Peak area %
1	17.1 ± 0.1	0.6 ± 0.3	17.1 ± 0.2	1.8 ± 0.1	17.1 ± 0.2	1.6 ± 0.1		17.0 ± 0.1	0.4 ± 0.1
2	23.6 ± 0.2	1.4 ± 1.7	23.7 ± 0.3	0.5 ± 0.1	23.7 ± 0.3	0.4 ± 0.1		23.5 ± 0.1	0.7 ± 0.5
3	25.8 ± 0.1	2.8 ± 4.1	25.8 ± 0.3	1.5 ± 0.1	25.8 ± 0.3	1.4 ± 0.1		26.0 ± 0.4	0.5 ± 0.3
4	27.0 ± 0.6	1.2 ± 1.1	26.5 ± 0.3	2.2 ± 0.1	26.5 ± 0.3	2.0 ± 0.2		27.2 ± 0.9	1.5 ± 1.3
5	28.2 ± 0.1	2.8 ± 0.7	28.1 ± 0.3	12.1 ± 0.6	28.2 ± 0.3	11.2 ± 0.8		28.4 ± 0.5	2.3 ± 2.2
6 (HHLL)	29.6 ± 0.1	**89.2 ± 7.0**	30.1 ± 0.3	**80.6 ± 1.0**	30.2 ± 0.4	**81.9 ± 1.2**		29.7 ± 0.1	**93.5 ± 2.4**
	Comparison of mean peak area (%) of all samples with that of cell bleed samples by Dunnett’s test (statistical significance)^c^								
Peak	15 ± 1		20 ± 1		25 ± 1				
1	NS		0.0001		0.0001				
2	NS		NS		NS				
3	NS		NS		NS				
4	NS		NS		NS				
5	NS		0.0001		0.0001				
6 (HHLL)	NS		0.0025		0.0056				

^a^Represent the average data from cell bleed samples collected at process intensification levels of 15 M cells/mL and 20 M cells/mL live cell densities

^b^Figure 1C shows the corresponding peaks identified on the expanded view of the electropherograms

^c^ANOVA showed significant *F*-statistic value (*p* < 0.005) for differences in area (%) for peak 1, 5, and 6. A comparison of means using Cell bleed values as control with Dunnett’s LSD showed either the peak area (%) values to be significantly different (*p* < 0.01) or not different (NS, not significant). Given values are ‘p’ values

### Effect of process intensities on mAb charge variants and distribution

**Fig. 2:**
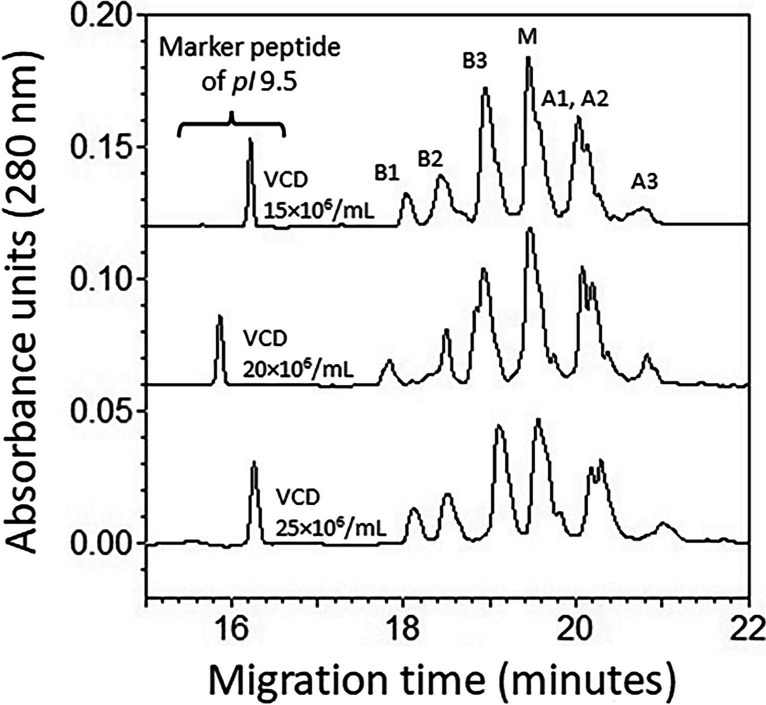
Representative profiles of the charge variants of the VRC01 mAb determined by cIEF analysis. The analysis used 3 M urea-gel as described in the methods. The peaks identified as B1, B2, and B3 correspond to the basic 1, basic 2, and basic 3 peaks, respectively. The peaks identified as A1, A2, and A3 correspond to the acidic 1, acidic 2 and acidic 3 peaks, respectively. The peak identified as M corresponds to the major peak while the sharp peaks on the left (around 16 min migration time) correspond to the pI 9.5 peptide internal standard. Quantitative analyses are presented in Table 3 and Table 4

The effect of increasing cell density on the charge variants of the mAb and protein isoelectric point (pI) was investigated by cIEF analysis. The cIEF profiles consistently showed 3 basic and 3 acidic peaks on either side of a central major peak (Fig. 2). The data in Table 3 show a summary of the charge variants that were observed under different cell densities. The data show that cell densities corresponding to different levels of process intensification affected two acidic peaks and the major peak, distinguishable by statistical analysis. The different charge variants’ contributions (individually toward the pI) were also analyzed by ANOVA (Table 4). The contribution of the peaks (peak area %) showed a small but distinct increment at higher cell densities for two basic peaks and the major peak as revealed by ANOVA. While the weighted average pI (of all peaks) of the mAb marginally increased with the cell densities (8.33 ± 0.09 at 15 × 10E06 cells/mL to 8.43 ± 0.09 at 25 × 10E06 cells/mL), an ANOVA performed on charge variants’ pI values, using the peak area (%) as weights, showed no significant differences (*F* statistic of 0.3 & *p* > 0.7). These data indicate that variation in cell densities during production process did not alter the amino acid composition of the mAb molecule that would result in a significant deviation in pI. As shown in Fig. 2, on an average, 3 acidic and 3 basic peaks can be identified around a major peak with a pI of 8.4 (± 0.1).

**Table 3:** Number of basic and acidic peaks, their pI and the extent of shift in the pI units with increase in cell densities and comparison of means after analysis of variance

Peaks^a^	Average pI (*n* ≥ 4) at different cell densities			Shift in pI of the peaks^b^		Analysis of Variance of peak values at 3 VCD^c^	
	VCD (10^6^·mL^−1^)			VCD (10^6^·mL^−1^)		*F*-value	*p* value
	15 ± 1	20 ± 1	25 ± 1	15 to 20	20 to 25		(VCD,10^6^·mL^−1^)^d^
Basic1-pI	8.86	8.87	8.90	0.01	0.03	2.693	NS
Basic2-pI	8.68	8.69	8.77	0.01	0.08	3.019	NS
Basic3-pI	8.51	8.52	8.58	0.01	0.06	3.733	NS
Major-pI	8.32	8.34	8.42	0.02	0.08	10.048	0.005 (25)
Acidic1-pI	8.13	8.13	8.23	0.00	0.10	16.618	0.001 (25)
Acidic2-pI	8.09	8.09	8.13	0.00	0.04	0.917	NS
Acidic3-pI	7.89	7.87	7.99	-0.02	0.12	21.129	0.001 (25)

^a^Peaks were manually identified, and the beginning and end time points were given for each peak for integration of area (Fig. 2) by 32 Karat software (AB Sciex)

^b^Positive values in the shift indicate an increase in pI and negative values indicate a decrease in pI

^c^The pI values of all peaks at the 3 different VCDs (3 groups) were analyzed (*n* = 4) by ANOVA followed by comparison of means by Dunnett’s test, where the group VCD at 15 × 10^6^·mL^−1^ was used as control

^d^The value in the parentheses indicates the VCD at which the mean peak was significantly different from the control (15 × 10^6^·mL^−1^); *NS*, not significant (*p* ≥ 0.05)

**Table 4:** Number of basic and acidic peaks and the peak area (%) values at three different cell densities and the comparison of means after analysis of variance

Peaks	Mean peak area (%) (*n* ≥ 4) at different VCD^a^ (10^6^·mL^−1^)			Analysis of variance & comparison of mean peak area (%) at 3 VCD^b^	
	15 ± 1	20 ± 1	25 ± 1	*F*-value	*p* value^c^
Basic1-pI	3.34 ± 0.19	3.80 ± 0.37	4.67 ± 0.34	4.638	0.027 (25)
Basic2-pI	7.21 ± 0.29	9.66 ± 1.23	7.84 ± 0.07	3.041	NS
Basic3-pI	18.76 ± 1.40	23.10 ± 0.87	19.52 ± 0.69	5.067	0.027 (20)
Major-pI	29.87 ± 1.00	28.98 ± 0.43	24.32 ± 1.61	7.067	0.012 (25)
Acidic1-pI	13.01 ± 1.83	9.56 ± 0.36	11.82 ± 2.75	0.834	NS
Acidic2-pI	10.38 ± 1.83	11.57 ± 1.87	10.45 ± 4.19	0.054	NS
Acidic3-pI	5.75 ± 1.08	4.38 ± 0.53	4.24 ± 0.42	1.284	NS

^a^Normalized peak area (%) values were determined by integration with 32 Karat software (AB Sciex) for the peaks identified manually the beginning and end timepoints for each cIEF profile (Fig. 2). The values given are mean ± standard error of mean

^b^The peak area (%) values of all peaks at 3 different VCDs (3 groups) were analyzed (*n* = 4) by ANOVA followed by comparison of means by Dunnett’s test, where the group VCD at 15 × 10^6^·mL^−1^ was used as control

^c^The value in the parentheses indicates the VCD at which the mean peak area (%) was significantly different from the control (15 × 10^6^·mL^−1^); *NS*, not significant (*p* ≥ 0.05)

### Effect of process intensities on mAb glycosylation and microheterogeneity

**Fig. 3:**
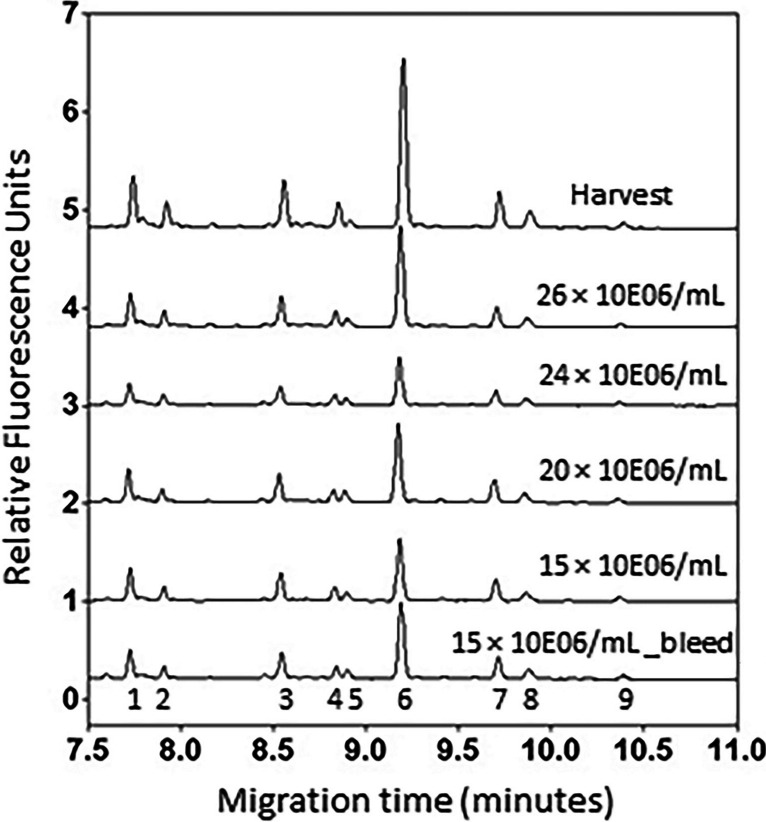
CE analysis of VRC01 mAb N-glycans labeled with APTS. Glycans were deglycosylated from 250 µg of mAb protein in parallel and labeled with APTS. The labeled glycans were then purified into water in parallel using magnetic beads and analyzed by CE with a 488 nm Laser Induced Fluorescence detector subjecting to a sequence analysis in CE under a single method. The representative CE profiles of APTS labeled N-glycans show that the type of glycans (peaks 1 through 9) did not vary at different levels of process intensification whereas their relative distribution varied. Quantitative results are presented in Table 5

**Fig. 4:**
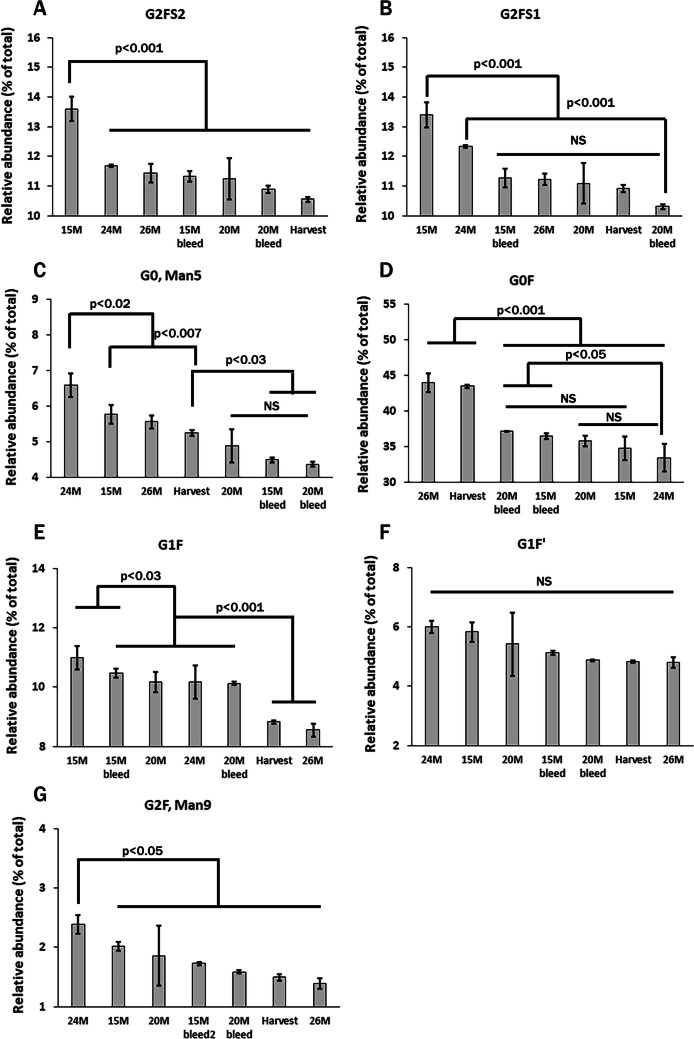
Relative abundance (peak area %) of glycan types of VRC01 mAb from bioreactor samples of different viable cell densities determined by CE analysis and their comparison. The samples (groups) included in the analysis were media harvested at viable cell densities of 15 × 10E06 cells/mL (15M), 20 × 10E06 cells/mL (20M), 24 × 10E06 cells/mL (24M), 26 × 10E06 cells/mL (26M), 15M cell bleed, 20M cell bleed and the final harvest (3 ≤ n ≤ 9). Peak area (%) values were obtained on the glycan elution profiles of CE separations by integrating the peaks using 32 Karat Software (AB Sciex) and were normalized to total peak area under all the peaks identified. Comparison of mean differences was performed by ANOVA, followed by a Tukey–Kramer’s HSD test. NS, not significant

Protein micro-heterogeneity may be affected by the extent of glycosylation and type of attached glycans. We investigated the variation in mAb glycan profile due to variation in cell densities during production. The results in Fig. 3 show the APTS labeled N-glycan profiles by CE analysis of whole mAb protein samples produced under varying cell densities. A comparison of N-glycan profiles from the whole mAb protein samples obtained under different VCD shows that variations in N-glycan types occurred at various process intensification levels. No additional peaks or a major shift in the migration time of the peaks were observed, however, the peak areas belonging to individual peaks showed differences. A comparison of peak areas (%) of glycan types of VRC01 mAb of different VCD is given in Fig. 4. The results of ANOVA show significant differences for the test statistic *F*-ratio (*p* < 0.05) (Table S2). Peak area (%) for each N-glycan elution profile was determined by integration using 32 Karat software. The peak area (%) values were compared for each N-glycan type of samples across all process intensifications. The data show that differences were statistically significant (*p* < 0.05) among different process intensities for each glycan type except G1F’ (Fig. 4F) as shown by the *F*-statistic. Pairwise comparison of mean values showed statistical differences (*p* < 0.05) between the groups. Since the glycan types contribute to the microheterogeneity in the produced mAb by way of their relative composition, these data indicate heterogeneous glycosylation of the mAb leading to varying microheterogeneity of the mAb under different process intensifications. While CE provided definitive data on the sialylated glycans (which eluted earlier than other glycan types) the HPLC analysis of the 2-AB labeled glycans gave better resolution for the different glycans, that gave about 13 identifiable peaks (based on the standards used; Figure S9). HPLC data on the glycan content corroborated the CE data by showing significant group differences for different type of glycans (Table S3). However, the extent of differences observed in the CE analysis is much greater than that seen in HPLC analysis, that could possibly be attributed to more sample variation (relatively higher coefficient of variation compared to the data in CE analysis) observed in HPLC data.

The influence of varying cell densities on the N-glycan type and content of the mAb were analyzed by a regression model such that relative abundance of glycan type, $y=mx+c+e$ wherein the viable cell density is the independent variable,* x*. Other parameters *m, c* and *e* represent the slope, intercept and the error associated, respectively. Statistics determined by the regression analysis of variance (RANOVA) of the data and their significance analysis are presented in Table 5. The peaks identified in Fig. 3 are the N-glycan types that are analyzed here. A significant *F*-ratio (low probability value) indicates a very low risk for the regression model and a significant *t*-ratio indicates that the risk is low to accept the slope is nonzero. Both the *F*-ratio and the *t*-ratio for the slope for four glycan types, namely, G2FS2, G2FS1, G0F, and G1F-Man7 were found to be statistically significant. G2FS2, G2FS1, and G1F-Man7 showed a negative non-zero slope and G0F showed a positive non-zero slope with viable cell density. However, other glycan types that include 2 unknown species and G0-Man5, G1F’ and G2F-Man9 showed an insignificant *F*-ratio and *t*-ratio indicating the slope was negligible.

**Table 5:** Regression analysis of variance for the relative abundance of glycan types at various cell densities determined by capillary electrophoresis^a^

Peak number^b^	N-glycan type	*F*-ratio	*p*-value	*t*-ratio for slope	*p*-value
1	G2FS2	79.8973	0.0001*	-8.94	0.0001*
2	Unknown 1	0.2901	0.5956	-0.54	0.5956
3	G2FS1	63.6800	0.0001*	-7.98	0.0001*
4	G0, Man5	0.0301	0.8638	0.17	0.8638
5	Unknown 2	0.1258	0.7267	-0.35	0.7267
6	G0F	31.6234	0.0001*	5.62	0.0001*
7	G1F, Man7	21.2395	0.0001*	-4.61	0.0001*
8	G1F’	0.3809	0.5434	-0.62	0.5434
9	G2F, Man9	0.0005	0.9818	0.02	0.9818

^a^The influence of viable cell density (VCD) on the N-glycan content (*y*) in the VRC01 mAb was analyzed using a model: $y=mx+c+e$, such that the VCD was the independent variable, *x*; *m, c* and *e* were the slope, intercept and the error associated, respectively. The probability for the *F*-ratio indicates the risk for the model and the probability for the *t*-ratio for slope indicates the risk to consider *m* ≠ 0. The data points included in the analysis, *n*, varied from 18 to 24 for different glycan types

^b^The peak numbers are those indicated in Fig. 3

*Indicates the risk is very low

Glycan analysis by HPLC technique also gave comparable results. Table 6 shows the regression analysis of the relative abundance (%) of the different glycan types determined in the VRC01 mAb by HPLC analysis. Analysis was performed on the N-glycans isolated from the whole mAb protein as well as from the isolated HC and LC polypeptides to understand if there were glycans specific to either LC or HC and in what way they were affected by varying process intensity. From the whole mAb, G2FS2 and G1F showed a negative non-zero slope whereas G0F showed a positive non-zero slope with increasing VCD. Other glycan types did not show statistically discernible slopes. Glycan analysis performed on the isolated LC and the HC of the mAb gave interesting results. G2FS2 and G2 type glycans were significant on the LC and either absent or below detection limits on the HC. Both G2FS2 & G2 from the LC showed a negative slope with the VCD by RANOVA. On the other hand, Man5, G0, G1 and G0F of the LC showed positive slope with VCD. Among the N-glycan isolated from HC, G1, Man5, and G0F showed positive slope with the VCD. Thus, the LC and HC appear to be differentially glycosylated and somewhat differentially affected with increasing process intensity.

**Table 6:** Regression analysis of variance for the relative abundance of glycan types at various cell densities determined by HPLC analysis^a^

	For whole mAb			For LC of the mAb			For HC of the mAb		
N-glycan type	*F*-ratio	*t*-ratio for slope	*p*-value	*F*-ratio	*t-*ratio for slope	*p*-value	*F*-ratio	*t*-ratio for slope	*p*-value
G2FS2	6.6624	-2.58	0.0170*	7.9591	-2.82	0.0144*	NM^b^	NM	NM
G2F	4.7831	2.19	0.0364*	3.0379	1.74	0.1005	1.6770	1.30	0.2178
G2^c^	2.3776	1.54	0.1374	31.9942	-5.66	0.0001*	NM	NM	NM
G1	3.2445	1.80	0.0854	22.46	4.74	0.0002*	27.1157	5.21	0.0004*
Man5	1.3030	1.14	0.2659	6.0091	2.45	0.0261*	14.2810	3.78	0.0016*
G0	0.1892	0.44	0.6678	6.0553	2.46	0.0256*	1.0726	-1.04	0.3134
G0F	76.5707	8.75	0.0001*	9.49	3.08	0.0072*	10.0287	3.17	0.0060*
G1F	16.8254	-4.10	0.0005*	0.1874	-0.43	0.6709	2.8192	-1.68	0.1095

^a^The relationship between VCD and the N-glycan content (*y*) in the VRC01 mAb was analyzed using a model: $y=mx+c+e$, such that the VCD was the independent variable, *x*; *m, c* and *e* were the slope, intercept and the error associated, respectively. The probability for the *F*-ratio and the *t*-ratio are given (common *p*-value). A significant *p* = value (*p* < 0.05) indicates the risk for the model is negligible and risk to consider *m* ≠ 0. The data points included in the analysis ‘n’ equaled to 15–33 for all the glycan types analyzed. * Indicates the risk is very low

^b^NM, not measured due to below quantification limit

^c^The relative abundance of G2 type glycan was less than 2% when analyzed from the whole mAb and there was no statistically discernible trend with VCD, whereas when analyzed from LC alone its relative abundance increased (from 4.7 to 8.7% in different samples) and showed negative slope with VCD

**Fig. 5:**
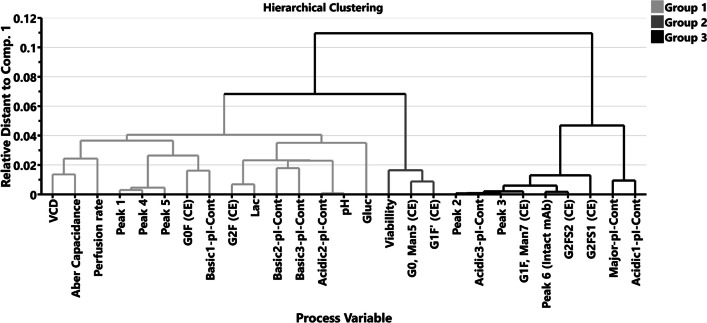
Hierarchical clustering of process variables. Hierarchical clustering of process variables groups determined by single linkage. Three major groups were formed, with groups 1 and 2 closer together when compared to group 3

The principal component analysis (PCA) model with three PCs accounted for > 90% of the model dataset with an *R*^2^ of 0.96 (Figure S13). However, Q^2^ for the model was ~ 0.39 that indicated that the model does not have the ability to predict the process intensification impact on PQAs outside our experimental dataset. Nevertheless, the PCA analysis provided correlative relationships among the different parameters. A hierarchical clustering analysis was also performed using a single linkage clustering following the PCA model (Fig. 5) using process parameters and measured product quality attributes. The hierarchical clusters are based on single linkage clustering and index sorting. Without having any bias towards the data sets, we performed cluster analysis to understand how closely the parameters are related to one another. Hierarchical clustering is an unsupervised type of machine learning wherein the algorithm searches for patterns in data sets with no pre-existing labels (Granato et al. 2018; Preud’homme G 2021). In a preliminary analysis, the metabolites glutamine, glutamate, ammonium, ions (Na, K, Ca), pCO_2_ and pO_2_, osmolality and cell diameter did not show interpretable correlations with the critical quality attributes measured on the mAb. Therefore, we excluded them from the single linkage clustering analysis, wherein the distance between a pair of clusters is the Euclidean distance between the two observations (one in each cluster) closest to each other. Parameters that are clustered in the same group (defined to have a distance < 0.05) and closer by distance to one another tend to be related and can also indicate a positive correlation. Conversely, further distanced parameters or groups are less related. However, these clustering of groups does not indicate whether a group or parameter will have a negative correlation. Hence, a correlation matrix was also utilized to better understand negative relationships between the process variables and measured parameters (Figure S14). The cluster analysis gave 3 groups of variables. Group 1 consisted of VCD, Aber Capacitance, perfusion rate, lactate (Lac), pH, glucose (Gluc), G0F, G2F, peak 1, 4, and 5 from the CE-SDS analysis under the non-reducing conditions, and the relative contributions of charge variant peaks of basic 1, basic 2, basic 3 and acidic 2. Group 2 consisted of viability, G0-Man5 and G1F’. Group 3 consisted of the relative contributions to charge variant peaks — acidic 3, major, acidic 1, peak 6 (intact mAb, HHLL), G2FS2, G2FS1 and G1F-Man7. Of note, the VCD was in group 1 and the mature type sialylated glycans (G2FS1, G2FS2) and G1F-Man7 were in group 3 and were separated. Similarly, VCD was separated from the intact mAb (HHLL) and the charge variant peaks, acidic 1, acidic 3, and major peak.

## Discussion

Therapeutic protein drugs possess specific molecular characteristics that are critical for the efficacy of the drugs in vivo. These characteristics are termed critical quality attributes (CQAs) (Alt et al. 2016; O'Flaherty et al. 2020). For mAbs, the molecular integrity, glycosylation, aggregation, and the state and extent of oxidation of amino acid residues have been shown to influence the in vivo function (Gupta et al. 2022; Higel et al. 2016; Krapp et al. 2003; Li et al. 2016) and hence considered critical quality attributes. Most of these CQAs are affected by how the manufacturing processes are performed, e.g., cell culture in bioreactors, purification, formulation, packaging and even storage and transportation of the mAb drugs. The manufacturing processes are optimized with a goal to maximize the production with a target product quality profile. This goal is achieved via optimization of cell culture media, addition of specific supplements, optimizing the bioreactor feeding regimen, better process control and genetic engineering of the host cell to increase the live cell density and in turn increase volumetric output (Li et al. 2010; O'Flaherty et al. 2020; Romanova et al. 2022). Although these strategies are common in fed batch production processes, in extended manufacturing processes removal of metabolic byproducts and replenishment of fresh nutrients are achieved by bioreactor perfusion with fresh medium, to attain high live cell densities. These process intensification strategies have resulted in significant improvement in productions (Clincke et al. 2013). However, these intensification strategies may impact product quality and therefore, it may be necessary to evaluate the impact of increasing volumetric output (via perfusion culture) on the CQAs of mAbs. The data presented in our work show the effect of different cell densities in perfusion culture on some important CQAs of the VRC01 mAb.

### Protein integrity under process intensification

The CE-SDS analysis under non-reducing conditions showed that protein integrity was affected by higher cell densities. The amounts of proteolytic enzymes may increase in the bioreactor broth at higher cell densities which in turn may affect protein integrity. Further, the cells may release enzymes under unknown physiological stresses which may also affect the integrity of produced recombinant protein. An increase in lactate levels was observed during the bioreactor run that indicated inefficient oxidation of glucose by excessive aerobic glycolysis, also known as the Warburg effect (Buchsteiner et al. 2018). Higher lactate levels may cause a decrease in the pH of intra and extracellular microenvironments (cf. Figure S11F, lactate profile during bioreactor run). Increased acidity could impact the physiology and viability of cells. The lower pH coupled with altered cell physiology and stress leading to release of endosomal enzymes such as gamma interferon-inducible lysosomal thiol-reductase (GILT) can cause reduction of disulfide bonds (Arunachalam et al. 2000). Surprisingly, we observed the highest protein integrity (%) in cell bleed samples, which may be ascribed to the limited exposure of the mAb to proteolytic and or disulfide bond reducing enzymes as the cells were removed by centrifugation immediately after cell bleed. It should be noted that the perfusion was started on day 3 and the process intensification samples were drawn from day 8 and later (Table 1).

Keeping the pyruvate dehydrogenase (PDH) in an active form by inactivating the PDH-kinase using dichloroacetate (DCA) may reduce lactate formation and maintain the cell microenvironment unaltered (Buchsteiner et al. 2018). Consequently, an increase in peak VCD and high viability was attained that led to approximately doubling of antibody titer production in prolonged fed-batch cultures (Buchsteiner et al. 2018). Integrating such controls in perfusion cultures may help in maintaining healthier cells even at higher cell densities which could result in better protein integrity.

The terminal glycan structures (e.g., galactose and sialic acids) on the proteins can sterically hinder the active sites in proteases and protect the amino acid residues adjacent to the glycosylated site (Solá and Griebenow 2009). A comparison of results in Fig. 4 showed that mAbs produced at a lower cell density of 15 × 10E06 cell/mL contained higher amount (~ 15%) of mature type glycan with sialic acid and galactose residues compared to mAbs produced at higher cell densities of 20 and 25 × 10E06 cell/mL (Fig. 4A, B and E). In our bioreactor run, the mAbs produced at lower cell density of 15 × 10E06 cell/mL, contained more intact molecules (~ 10%) than the mAbs produced at higher cell densities of 20 and 25 × 10E06 cell/mL (Table 2).

Our analysis of charge variants of VRC01 mAb with the help of cIEF indicated 3 acidic and 3 basic peaks around a major peak with a wide pI for the VRC01 mAb (8.4 ± 0.4). The marginal variations seen in pI of the mAb peaks (cf. Table 3) indicated that, overall, no significant modifications occurred on the charged residues of the protein backbone. However, a small basic shift in the pI was noticeable with the increasing process intensification (Table 3). The relative contribution of the charge variants (peak area %) also appeared to change with the process intensification (Table 4). The ANOVA performed on the peak area (%) of the charge variants at the three VCDs showed that the basic peak 1, basic peak 3 and the major peak tended to deviate significantly at higher cell densities compared to 15 × 10E06 cells/mL. These data appear to be consistent with the negative non-zero slope observed for the sialylated glycans with the VCD (Table 5 and Table 6). The affected physiology of the cell due to variation in the microenvironment may have influenced the recombinantly produced protein to marginally vary the pI, due to minor modifications in the molecular (structural) conformations (Loell and Nanda 2018). In this context, marginal shifts seen in pI for the purified VRC01 mAb from a process intensity of 15 × 10E06 cells/mL to higher intensities can be reconciled with an increase in lactate concentration (Figure S11F) in the medium (from 15 to 20 × 10E06 cells/mL) that points toward an acidic shift in the microenvironment of the cells.

Other investigators have focused on pH stability of the protein and the subcellular pH rather than the average pI of the protein, wherein net charge at the environmental pH has been thought to mitigate protein aggregation and maintain protein activities (Chan et al. 2006; Garcia-Moreno 2009; Talley and Alexov 2010). Therefore, the influence of bioprocess cell culture medium pH on the protein molecular integrity, and activity appears to be complex. Perfusion culture has been developed to (a) precisely control the pH and reduce the impact of metabolic byproducts such as lactate and ammonium; (b) is thought to limit exposure to extracellular enzymes (e.g., proteases, glycosidases, disulfide reductases) and limit changes in environmental conditions (pH, temperature) that can degrade the protein drug substance. The data on the pI of VRC01 mAb underscores that a focus on pI analysis appears to provide direct information on the mAb expression fidelity of the cells and the extent of stability afforded by the process against modifications of the charged residues of the protein backbone. Furthermore, the overall direction of marginal shifts in the pI of the protein can also provide a basis for better selection of the formulation buffer pH and the buffer salt.

Influence of varying viable cell densities (VCD) on the glycan type and content of the VRC01 mAb was analyzed by a regression model and regression analysis of variance (RANOVA). Interestingly, mature type glycans of the whole mAb analyzed by CE, namely G2FS2, G2FS1 and G1F-Man7, gave a negative slope. On the contrary, G0F showed a positive slope with VCD (Table 5). Trappe and co-workers have reported similar findings for variation in the charge variants profile and the degree of sialylation, oligomannoses, and terminal galactose of cetuximab produced from HEK293 and CHO cells (Trappe et al. 2022). However, G0-Man5, G1F’ and G2F-Man9 type showed negligible slopes with VCD. The multivariate data analysis using PCA and cluster analysis corroborated the RANOVA, in the sense that VCD and the mature type (sialylated) glycans were found to be in separate groups (Fig. 5). Interestingly, in a correlation matrix, VCD was found to have negative correlation values with respect to the mature type glycans, major peak area (%), and acidic peak 3 area (%) of the charge variants and the intact molecular content (HHLL) of the mAb (Figure S14). On the other hand, the mature type glycans (G2FS2 and G2FS1) had positive correlation values with intact molecular content (HHLL) of the mAb. A past report noted that a correlation exists between mature glycan content and molecular integrity of the proteins as the mature type glycans (with terminal galactose and sialic acid residues) can protect the adjacent amino acids on the target proteins from the active sites of proteases (Solá and Griebenow 2009). These data suggest that media supplementation may be required to maintain the desired complexity of N-linked glycan profiles at longer culture duration and higher cell densities (Radhakrishnan et al. 2017; Sha and Yoon 2019). Acidic charge variants have resulted in higher CDC (complement-dependent cytotoxicity) activity (Yang et al. 2014) and the levels of different terminal sugar residues have impacted CDC and ADCC (antibody-dependent cell-mediated cytotoxicity) activity (Grainger and James 2013; Wang et al. 2022; Yang et al. 2014).

### Variation in N-glycan types and protein homogeneity under process intensification

N-glycan analysis of the VRC01 mAb was performed by two orthogonal techniques: (a) labeling the N-glycans with APTS and analysis by CE, and (b) labeling the N-glycans with 2-AB and analysis by HPLC. Both gave comparable results. Comparison of different glycan types for their relative abundance showed statistically significant differences in samples across different cell densities, cell bleed samples and harvest (Fig. 4). The data suggest that the cell density variation has specifically affected sialylated glycans, G1F, G0-Man5, and G0F type glycans. In this regard, the complexity of glycans seemed to affect how the glycan contents would vary with process intensification. Hybrid and more complex glycans contain subsequent N-acetyl glucosamines on the core mannoses. The actions of N-acetylglucosyl transferases facilitate further addition of galactoses and sialic acid on the N-acetyl glucosamines leading to the formation of extended “antennae” that we term as more mature type glycans. These mature type glycans (galactosylated and sialylated) gave a negative slope with VCD. An earlier report showed somewhat similar results with respect to galactosylated glycan type (Mellahi et al. 2019). These authors reported that harvesting at a lower cell density resulted in an N-linked glycan profile that had higher galactosylated glycans when compared to the higher cell density harvests in their fed-perfusion culture. While our studies confirmed the results with respect to mono-galactosylation (G1F), the G2F species gave a negligible slope with VCD. These data suggest that in the (VRC01) CHO cell line, the mAb produced was less susceptible to process intensification for G2F glycan type as compared to other fucosylated glycan types (G0F and G1F), high mannose and sialylated glycan type under perfusion culture and increased cell densities. The decrease in more complex N-linked glycan profiles could have been a result of altering concentrations in nucleotide-sugar precursor, amino acids, and trace metals in the culture medium as the process intensified during production process (Chen et al. 2019). In this regard, it is noticeable that zinc supplementation improved the cell viability, production and harvest purity of the lysosomal enzyme, β-glucuronidase, by suppression of apoptosis in another CHO-K1 cell line (Graham et al. 2020). Furthermore, a variation in zinc concentration affected fucosylation levels of this lysosomal enzyme N-glycans under fed-batch cultures (Graham et al. 2021). On the contrary, Lipscomb and coworkers observed more sialylated protein under perfusion culture that could possibly be attributed to the slower cell growth, which favored producing glycosylated proteins with mature glycan types (Lipscomb et al. 2004).

Furthermore, the glycans associated with the LC and HC polypeptides of the VRC01 mAb analyzed by HPLC provided interesting results, which complemented and corroborated the observed effect of the process intensification on the N-glycans of the whole mAb protein. One notable observation was that the sialylated glycan type and the G2 glycan types were found on the LC polypeptide and not quantifiable from the HC polypeptide. Notably, the relative abundance of G2 type glycan was less than 2% when analyzed from the whole mAb and there was no trend with VCD, whereas when analyzed from LC alone, its relative abundance increased (from 4.7 to 8.7% in different samples) and showed negative slope with VCD. More LC glycans (G2FS2, G2, Man5, G0, G1 and G0F) showed statistically discernible non-zero slope than the HC glycans (G1, Man5, and G0F) with process intensification (Table 6).

In this work, we measured sialylated N-linked glycans from the mAb samples produced by the (VRC01) CHO cells, but the sialylated N-linked glycan type had a negative slope with VCD (Table 5 and Table 6). Sialylated N-linked glycans are important and a CQA of the mAb products as they affect serum half-life, clearance, and immunogenicity (Bhide and Colley 2017; Bork et al. 2009). Furthermore, heterogeneity is often observed for sialylated N-linked glycan due to variation in process affecting cell physiology. In a critical study evaluating the role of specific glycoforms of the mAb, glycoengineering and glycoform-resolved pharmacokinetics (PK) measurements were made in a rat model using four differentially glycoengineered mAbs (Falck et al. 2020). The mass-spectrometry analysis of the glycoforms of the mAb in the rat blood showed high clearance for Man5 and hybrid-type (Man5G0) glycoforms. However, monoantennary and diantennary glycoforms of the mAb that have only core three mannoses were found to clear more slowly (Falck et al. 2020). Hence, careful consideration may be given for supplementations of a perfusion culture as a next step to better understand and overcome the limitations of process intensification and achieve higher levels of mono and di-antennary, galactosylated and sialylated N-linked glycosylation and better protein integrity.

Overall, these data indicate that changing production processes with the goal to either improve production or process consistency or both can alter the micro-heterogeneity of the products. Therefore, comparability studies may be performed to confirm that changes in microheterogeneity, arising from variation in the N-glycan type and composition, result in comparable in vivo activities and thereby similar efficacy, before and after the effected change in the production process. Furthermore, when a batch process is changed to an extended production process or continuous manufacturing process, it becomes necessary to carefully monitor the process. The process intensification can produce heterogeneous product (from the glycosylation perspective), and appropriate lot allocations are critical to minimize variation. In addition, determining what extent of process deviation from the steady state alters the product quality attributes and contribute to the altered product performance may be necessary.

The focus of our studies was the effect of process intensification on the PQAs under perfusion culture. Since N-glycosylation is an important PQA and most susceptible due to process variations, it was analyzed by two techniques. However, one of the limitations of the studies is the absence of a direct N-glycan analysis on the mAb. Mass spectral analysis of the intact protein can directly reveal the LC and HC associated N-glycan types and their relative content (Shrivastava et al. 2022). However, the mass spectrometry analysis cannot distinguish between isobaric structures (G1F and G1F’). It was also reported that Man5 and G0-N co-elute/overlap with G0 and G1 forms (Sinha et al. 2008), and thus separation on HPLC or UPLC systems with labeled N-glycans becomes necessary. Second, a direct cause for the reduction in the molecular integrity of the mAb protein in terms of a physiological effect is not confirmed by measuring the gamma interferon-inducible lysosomal thiol-reductase (GILT), which was not the focus of the study. Third, quantification of titer and recoveries after purification of the mAb were found to be less relevant as they would not influence the results of the effect of process intensification on product quality attributes under the perfusion culture. Therefore, they were not documented.

In our studies, we investigated the impact of process intensification in a perfusion cell culture on PQAs such as integrity, pI, and extent of N-glycosylation of an IgG1-κ monoclonal antibody. In the present study, CE-SDS data showed that better molecular integrity (89% intact mAb) was observed at lower cell density (15 × 10E06 cells/mL) while lower integrity (~ 80% intact mAb) was seen at 20 and 25 × 10E06 cells/mL. We hypothesize that decreased protein integrity at increased process intensification level could be a result of (i) altered environmental pH because of increased lactate due to the Warburg effect, leading to exposure of the mAb to thiol reducing enzymes and (ii) decreased protection against proteolytic action due to decreased mature type glycan content. The VRC01 mAb showed a major peak pI at basic pI (~ 8.4 ± 0.1), with identifiable 3 basic and 3 acidic peaks. The mAb protein showed a pI at 8.4 ± 0.4 with marginal variation across different process intensification levels, consistent with the variation in sialylated glycan types, indicating the process afforded high molecular expression fidelity and stability against modifications/substitution of the charged amino acid residues of the protein backbone. However, the distribution of content of glycan types differed statistically in the purified VRC01 mAb across different process intensity levels. More mature glycosylation (particularly sialylation) showed a negative slope with process intensification levels as seen by glycan analyses by both CE and HPLC. While N-linked glycans afford more stability for the protein, it appears that more mature galactosylated and sialylated N-linked glycans, and protein integrity are traded off for volumetric productivity at higher process intensifications. Therefore, studies on media additives can guide process intensification efforts to steer the CQAs favorably with volumetric production under perfusion culture of CHO cells.

## Supplementary Information

Below is the link to the electronic supplementary material.

Supplementary file1 (PDF 3591 KB)

## Data Availability

All data utilized to draw inferences are depicted in the form of Tables and Figures and in the supplementary material. Original source data such as HPLC runs and CE runs are within the institutional data repositories and can be made available when required.

## Declarations

### Disclaimer

This article reflects the views of the authors and should not be construed to represent FDA’s views or policies.
