# Supplementary material for: Effects of process intensification on homogeneity of an IgG1:κ monoclonal antibody during perfusion culture
Source: Appl Microbiol Biotechnol. 2024 Mar 26;108:274. doi: 10.1007/s00253-024-13110-9 (PMC10965650; doi:10.1007/s00253-024-13110-9)
Supplement: Supplementary file 1 [file 253_2024_13110_MOESM1_ESM.pdf]

Supplementary material to MS: No. AMBB-D-23-00405

Journal: Applied Microbiology and Biotechnology

Effects of process intensification on homogeneity of an IgG1:κ monoclonal antibody during perfusion culture

Authors: George Liang[1,2,3], Chikkathur N. Madhavarao[1,3,*], Caitlin Morris[1,2], Thomas O'Connor[1], Muhammad Ashraf[1], Seongkyu Yoon[2]

[1] Division of Product Quality Research, OTR/OPQ/CDER/FDA, Silver Spring, MD

[2] Department of Chemical Engineering, University of Massachusetts Lowell, Lowell, MA

[3] Contributed equally.

* Corresponding author

chikkathur.madhavarao@fda.hhs.gov

Disclaimer: This article reflects the views of the authors and should not be construed to represent FDA's views or policies.

Acknowledgements: The authors gratefully acknowledge the receipt of VRC01 CHO-K1 cell line produced by the Vaccine Research Center, NIAID, NIH, Gaithersburg, MD, USA. George Liang and Caitlin Morris were supported in part by the Oak Ridge Association of Universities (ORAU) and the Oak Ridge Institute of Science and Education (ORISE), through an agreement between the U.S. Department of Energy and the U.S. Food and Drug Administration. The authors thank David Keire and Xiaoming Xu for comments and suggestions and Ahmed Zidan for discussions.

Supplementary documentation

Table S1. Protein recovery data from the size exclusion choromatography after treating the VRC01 mAb with 2-, 5- and 10 mM DTT [a]

Sample	Protein Yield (µg)	Protein Sum (µg)	% Yield AUC	% SEC Recovery
10 mM DTT - 75kDa - Peak 1 (~75 kDa)	146.62		24.85	
10 mM DTT - 50 kDa - Peak 2 (~50 kDa)	49.56	590.01	8.40	59.001
10 mM DTT - 50 kDa - Peak 2.5 (~50 kDa)	137.82		23.36	
10 mM DTT - 25 kDa - Peak 3 (~25 kD)	256.01		43.39	
5 mM DTT - 75kDa - Peak 1 (~75 kDa)	175.35		20.57	
5 mM DTT - 50 kDa - Peak 2 (~50 kDa)	505.18	852.48	59.26	85.248
5 mM DTT - 25 kDa - Peak 3 (~25 kDa)	171.95		20.17	
2 mM DTT - 75kDa - Peak 1 (~75 kDa)	191.75		43.75	
2 mM DTT - 50 kDa - Peak 2 [b]	-	438.25	-	43.825
2 mM DTT - 50 kDa - Peak 2.5 (~50 kDa)	179.14		40.88	
2 mM DTT - 25 kDa - Peak 3 (~25 kDa)	67.36		15.37	

[a] Purified VRC01 mAb protein was treated with the three concentrations of DTT (dithiothreitol) as described in the methods section for separating the light chains (LC) and heavy chain (HC) polypeptides. Then size exclusion chromatography (SEC) was performed using a Superdex®200 Increase HiScale® 26/40 column on an AKTA avant 25 chromatography system as described in the methods section (also see Figure S3). Protein recoveries were determined after estimating the protein concentration in the fractions corresponding to the elution peaks (1, 2, 2.5 and 3) by BCA protein estimation assay.

[b] Not detectable (ND)

Table S2. A comparison of peak area (%) of glycan types of VRC01 mAb of different viable cell densities determined by CE analysis[a]

		Peak area (%)[c]		Comparison of group means by Tukey's HSD
**Peak number[b]**	**N-glycan type**	**F-value**	**p-value**	**Groups that are significantly different ($p<0.05$)**
1	G2FS2	22.89	0.0001	15M & 20M; 15M &24M; 15M & 26M, 15M & 15M bleed; 15M & 20M bleed
2	Unknown 1	4.95	0.0022	15M & 20M; 20M & 24M
3	G2FS1	13.73	0.0001	15M & 20M; 15M & 26M; 15M & 15M bleed; 15M & 20M bleed; 15M & harvest; 24M & 20M bleed
4	G0, Man5	20.28	0.0001	15M & 20M; 15M & 24M; 15M & 20M bleed; 15M & 15M bleed;15M bleed & harvest; 20M & 24M; 24M & 26M; 24M & 15M bleed; 24M & harvest; harvest & 20M bleed
5	Unknown 2	18.60	0.0001	15M & 20M; 15M & harvest; 15M bleed & harvest; 20M & 26M; 20M bleed & harvest; 20M & harvest; 24M & harvest; 26M & harvest;20M bleed & 26M
6	G0F	46.38	0.0046	15M & 26M; 15M & harvest; 15M bleed & 26M; 15M bleed & harvest; 15M bleed & 24M; 20M & 26M; 20M & harvest; 24M & 26M; 24M & harvest; 20M bleed & 26M; 20M bleed & harvest; 20M bleed & 24M
7	G1F, Man7	26.28	0.0001	15M & 20M; 15M & 24M; 15M & 26M; 15M & 20M bleed; 15M & 15M bleed;15M & harvest; 20M & 26M; 20M & harvest; 24M & 26M; 24M & harvest; harvest & 20M bleed; 20M bleed and 26M
8	G1F'	2.04	0.1018	NS
9	G2F, Man9	4.13	0.0058	24M & 26M; 24M & 20M bleed; 24M & harvest

[a] The samples (groups) included in the analysis were media harvested at viable cell densities of 15 × 10E06 cells/mL (15M), 20 × 10E06 cells/mL (20M), 24 × 10E06 cells/mL (24M), 26 × 10E06 cells/mL (26M), 15M cell bleed, 20M cell bleed and the final harvest ($3 \leq n \leq 9$)

[b] The peak numbers correspond to the identified peaks in Fig 3 (main text) and ascribed the N-glycan types based on the co-migration of glycan standards as shown in Figure S8.

[c] Peak area (%) values were obtained on the glycan elution profiles of CE separations by integrating the peaks using 32 Karat Software (AB Sciex) and were normalized to total peak area under all the peaks identified. Comparison of mean differences was performed by ANOVA, followed by a Tukey-Kramer's HSD test.

Table S3. A comparison of peak area (%) of glycan types of VRC01 mAb of different viable cell densities determined by HPLC analysis [a]

	Peak area (%) [b]		Comparison of group means by Tukey's HSD
**N-glycan type**	**F-value**	**p-value**	**Groups that are significantly different (p<0.05)**
G2FS2	4.04	0.0089	15M & Harvest; 15M & Bleeds
G2F	6.59	0.0006	20M & Bleeds; 24M & Bleeds; 24M & 26M; 24M & harvest
G2	7.23	0.0010	15M & harvest; 24M and harvest; 26 M & harvest
G1F	5.54	0.0017	15M & Bleeds; 20M & Bleeds; 24M & Bleeds
G0F	6.09	0.0010	15M & Bleeds; 20M & Bleeds; 24M & Bleeds; 26M & Bleeds; Harvest & Bleeds
Man5	2.95	0.0334	NS
G0	3.66	0.0139	26M & Bleeds
G1	5.65	0.0015	24M & Bleeds; 26M & Bleeds

[a] The samples (groups) included in the analysis were media harvested at viable cell densities of 15 × 10E06 cells/mL (15M), 20 × 10E06 cells/mL (20M), 24 × 10E06 cells/mL (24M), 26 × 10E06 cells/mL (26M), 15M cell bleed, 20M cell bleed and the final harvest ($3 \leq n \leq 9$)

[b] Peak area (%) values were obtained on the glycan elution profiles of HPLC separations by integrating the peaks using OpenLab CDS software (Agilent, Sta Clara, CA) and were normalized to total peak area under all the peaks identified. Comparison of mean differences was performed by ANOVA, followed by a Tukey-Kramer's HSD test using JMP software (Ver 16.0.0).

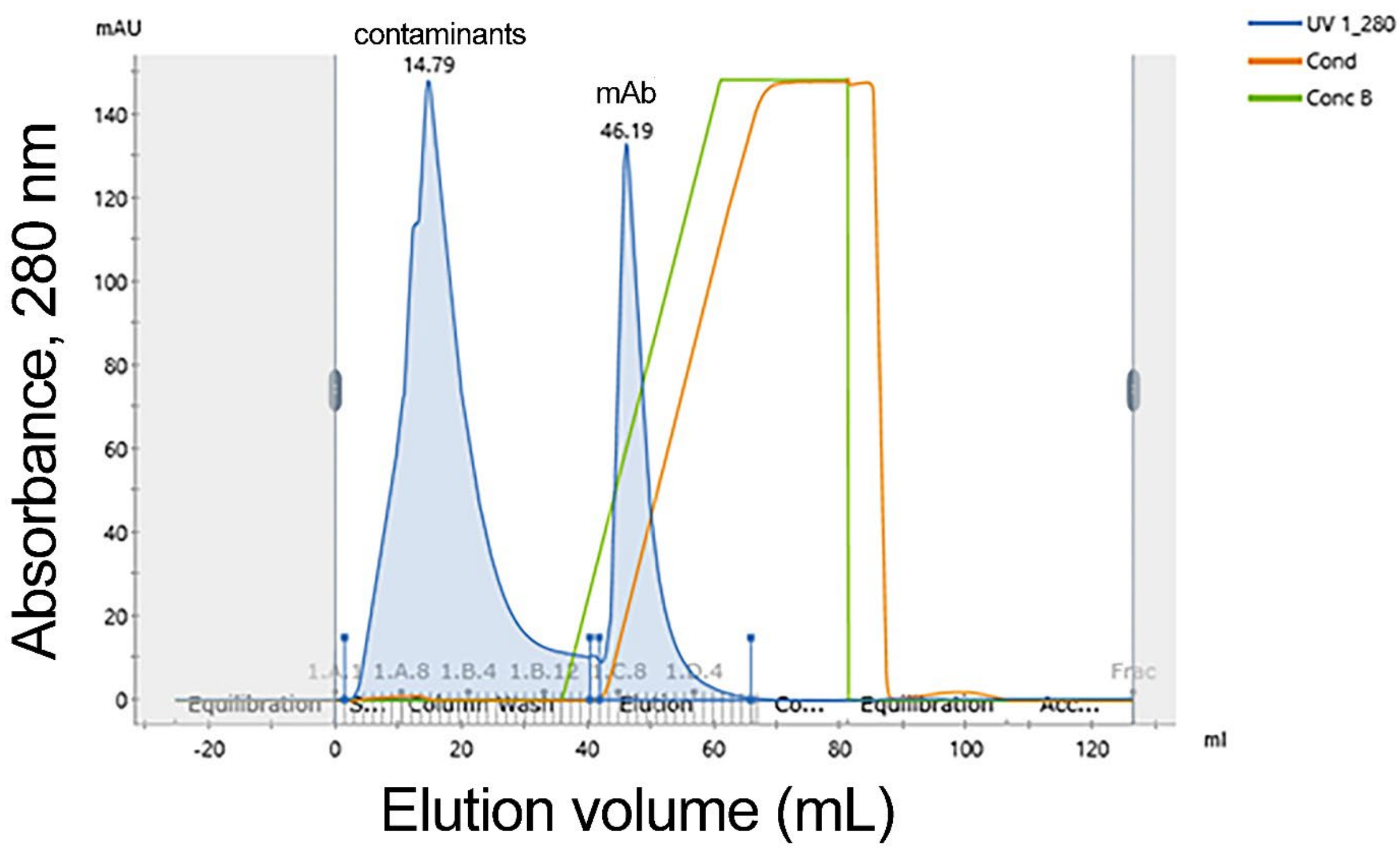

Figure S1. Elution profile from the DEAE chromatography. Only one peak corresponding to the mAb was eluted during the gradient elution from the DEAE column (from 0- 200 mM NaCl). Most of the mAb eluted by 75% of the gradient (≤ 150 mM NaCl) and no other peak was detected at 100% of elution buffer, in the next 8 column volumes. Contaminant proteins were removed in the flow through and during column wash.

A

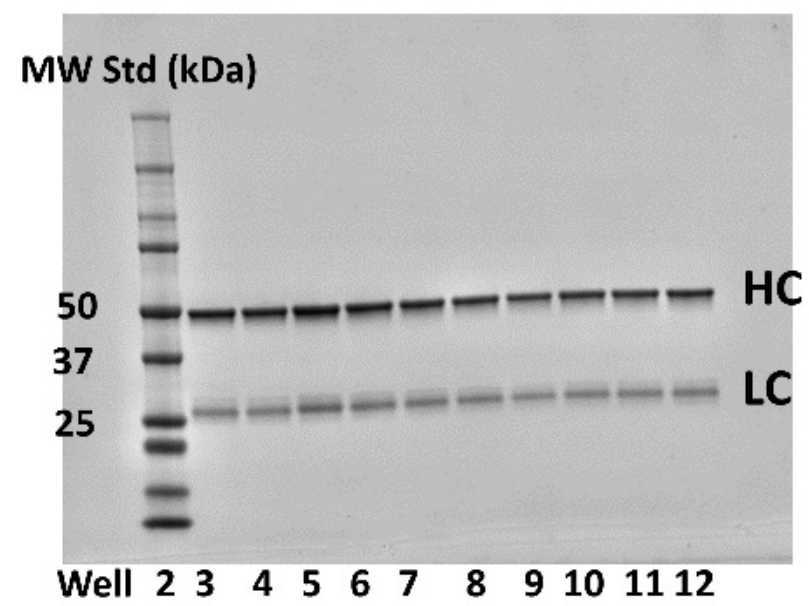

Well	Sample
2	Ladder
3	15 M data point 1
4	15 M data point 2
5	15 M data point 3
6	20 M data point 1
7	20 M data point 2
8	20 M data point 3
9	25 M data point 1
10	25 M data point 2
11	Harvest data point 1
12	Harvest data point 2

B

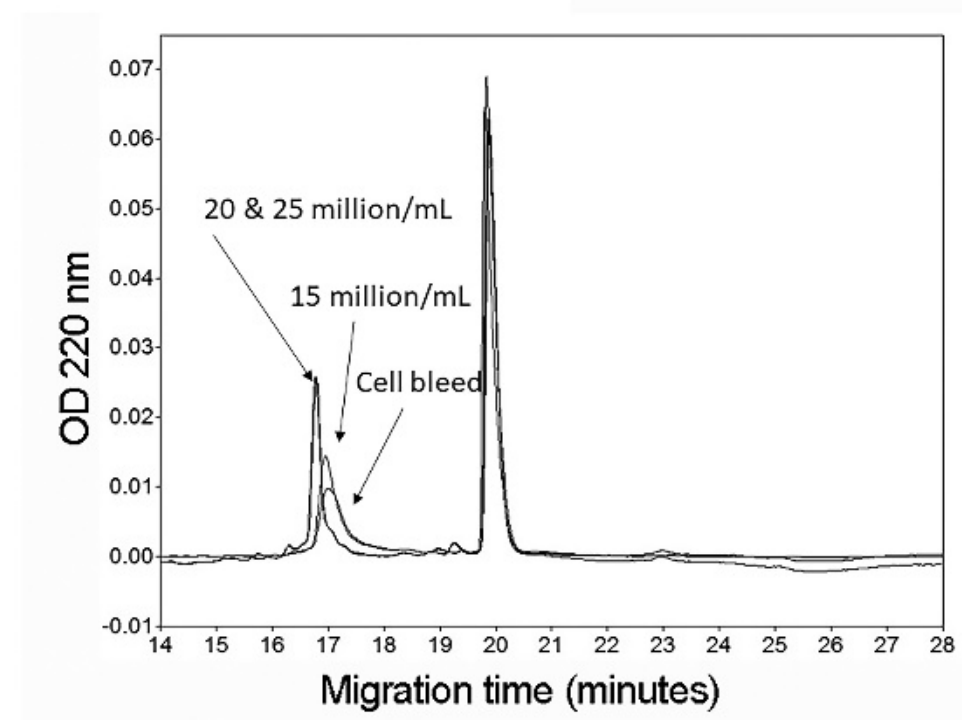

C

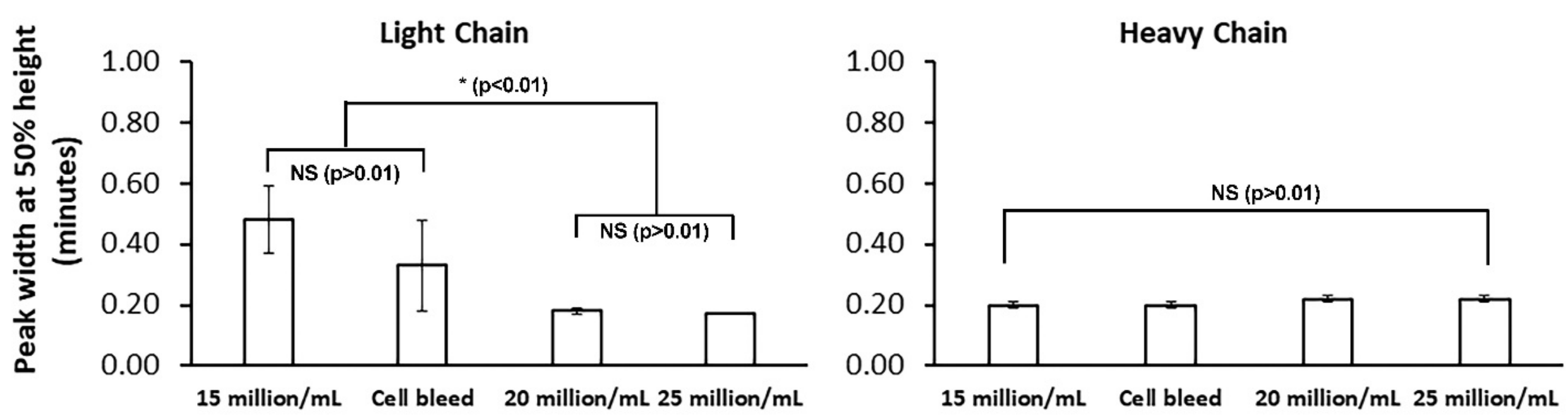

Figure S2. (A) SDS PAGE of the protein after protein A purification and anion exchange chromatography. (B) CE-SDS analysis under reducing conditions showing light chain (LC) molecular heterogeneity among different samples. Notice that the heavy chain (HC) does not show such variability. (C) Quantitative comparison of the peak width of LC at 50% height shows statistically significant differences ($p<0.01$) by Dunnett's t-test, but not the HC. Non-significance (NS) is indicated as NS ($p>0.01$).

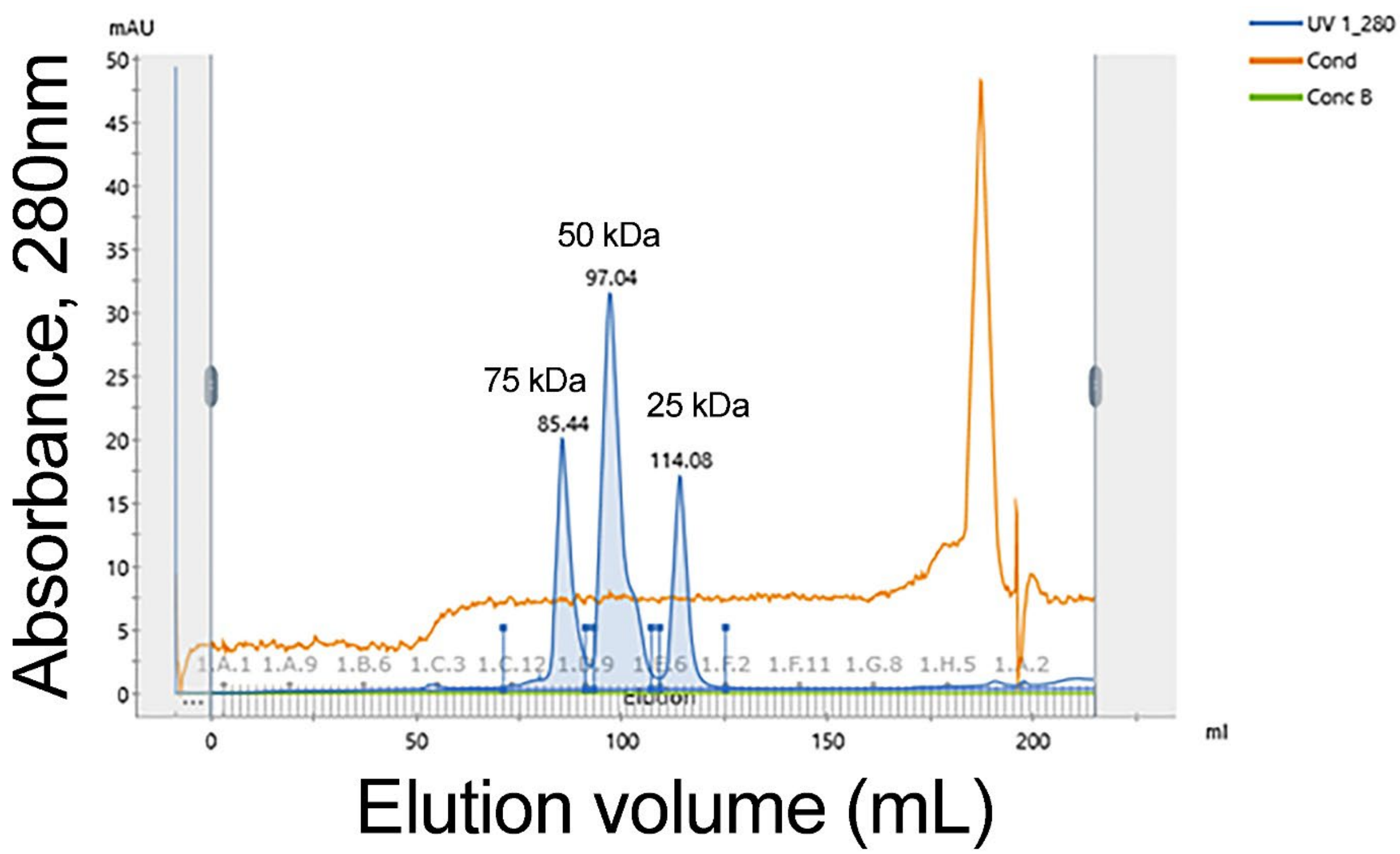

Figure S3. Elution profile from the size exclusion chromatography performed on Akta avant 25 purification system. The column Superdex®200 Increase HiScale® 26/40 was pre-washed with Milli-Q water (18.2 MΩ), followed by pre-equilibration (1 CV) with running buffer consisted of 0.05% SDS, 50 mM Tris-HCl, 1 mM DTT, 50 mM NaCl, pH 9.0. Elution was performed at a flow rate of 3.0 mL/min for 212 mL and 2mL fractions were collected into a deep well 96-well plate (at 6 ^{o}C). The fractions corresponding to the 50 kDa and 25 kDa peaks (Supplementary Fig A4) were concentrated separately for analysis.

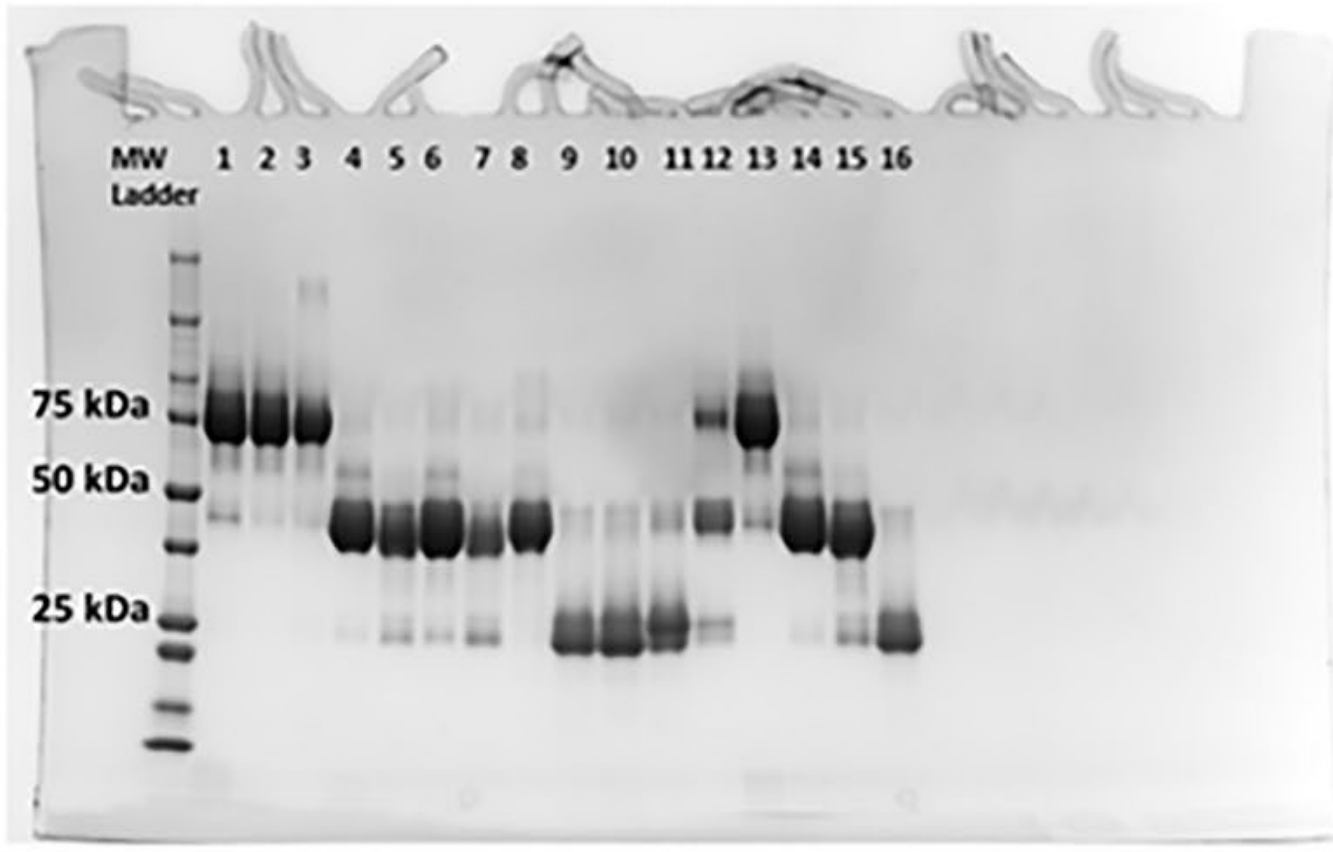

Well Number	Sample Name
1	2 mM DTT - 75 kDa (peak 1)
2	5 mM DTT - 75 kDa (peak 1)
3	10 mM DTT - 75 kDa (peak 1)
4	2 mM DTT - 50 kDa (peak 2)
5	2 mM DTT - 50 kDa (peak 2.5)
6	5 mM DTT - 50 kDa (peak 2)
7	10 mM DTT - 50 kDa (peak 2)
8	10 mM DTT - 50 kDa (peak 2.5)
9	2 mM DTT - 25 kDa (peak 3)
10	5 mM DTT - 25 kDa (peak3)
11	10 mM DTT - 25 kDa (peak 3)
12	2 mM original no SEC processed
13	2 mM DTT - 75 kDa (peak 1)
14	2 mM DTT - 50 kDa (peak 2)
15	2 mM DTT - 50 kDa (peak 2.5)
16	2 mM DTT - 25 kDa (peak 3)

Figure S4. SDS-PAGE analysis of the three peaks separated on size exclusion chromatography for isolating the light chain (LC) and heavy chain (HC) polypeptides from the VRC01 mAb. SDS-PAGE was performed on BioRad supplied 4-12 % gradient gel. The SDS-PAGE analysis showed the three peaks of SEC contained the isolated LC (~25 kD), HC (~50 kD) polypeptides and the peak that contained the partially reduced and separated mAb protein (~75kD).

A

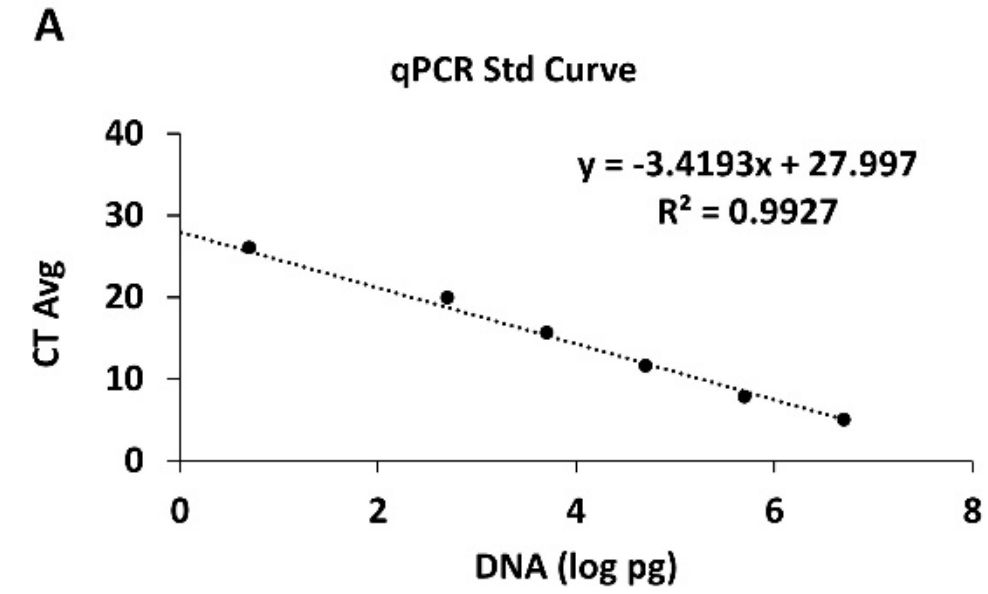

B

Sample Name	DNA (pg)
No Template Control (NTC)	0.8
VRC01-Reference Standard (RS) 1 (No DEAE Column)	0.3
VRC01-RS 1 - Pool 1 - I (DEAE Column)	Undetermined
VRC01-RS 1 - Pool 1 - II (DEAE Column)	Undetermined

Figure S5. (A) qPCR Standard Curve and (B) sample data with level of DNA contamination as determined by qPCR. Average number of cycles increased with decreasing level of DNA contamination. The standard curve has an R^2 of 0.99. Samples prior to DEAE column chromatography (NTC and VRC01 – RS 1), had 0.3-0.8 pg of DNA/mg of the protein and post DEAE purification (VRC01 RS 1 – Pool 1&2) DNA contamination level was lower than the detection limit of qPCR.

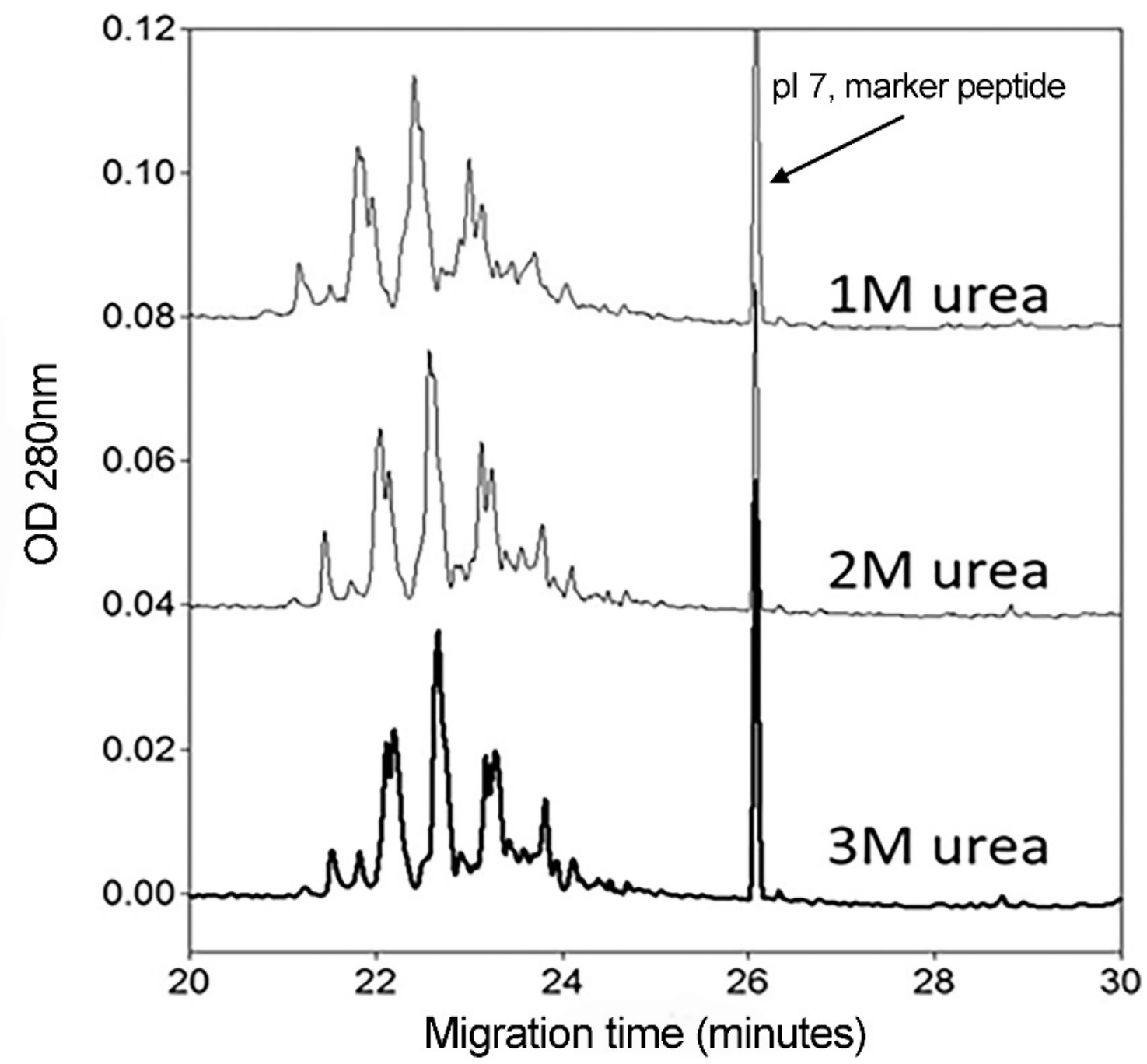

Figure S6. Standardization of cIEF conditions. The mAb was solvated in cIEF gel containing 1−3 M urea and based on the results, cIEF gel with 3 M urea was used for analysis of all the samples. Ampholytes with a broad pH range of 3−10 were used for separation and peptides with pI 7.0 and 9.5 were used as flanking markers with every sample. A standard curve was constructed (n=20) with 5 pI marker peptides (10, 9.5, 7, 5.5, 4.1) with a good correlation (r^2>0.99) for calculating the pI of the sample peaks. The sharp peak at ~26 minutes corresponds to marker peptide of pI 7.0.

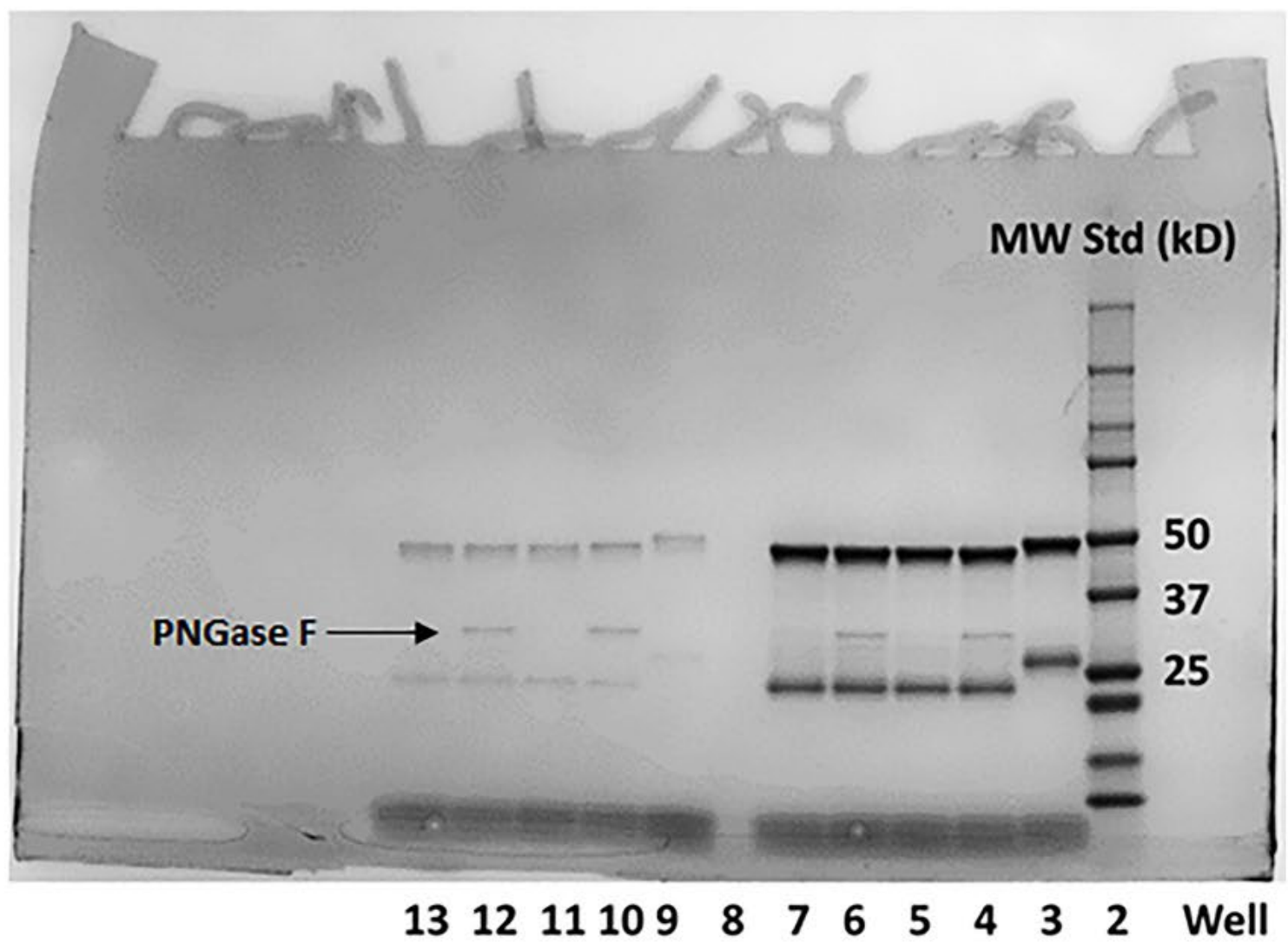

Well	Sample
2	Ladder
3	IgG Ref. – Control (no enzyme)
4	IgG Ref. – 2-hour Enzyme treatment
5	IgG Ref. – 2-hour Enzyme (1/5) treatment
6	IgG Ref. – 1-hour Enzyme treatment
7	IgG Ref. – 1-hour Enzyme (1/5) treatment
8	Blank
9	VRC01 – Control (no enzyme)
10	VRC01– 2-hour Enzyme treatment
11	VRC01– 2-hour Enzyme (1/5) treatment
12	VRC01– 1-hour Enzyme treatment
13	VRC01– 1-hour Enzyme (1/5) treatment

Figure S7. SDS PAGE analysis to ascertain the effect of deglycosylation of the mAb by PNGase-F enzyme treatment. IgG Ref is the reference standard of the VRC01 mAb produced by a batch culture. Lanes 3 and 9 of the SDS-PAGE image show the samples that were not treated with PNGase-F, and lanes 4-7 and 10-13, show the samples after treating with PNGase-F. A downward shift in molecular weight of both heavy and light chains are discernible. No differences between a 1 hour or a 2-hour treatment as well as 5X diluted or undiluted PNGase-F enzyme were seen. The arrow on the gel image shows the band corresponding to PNGase F.

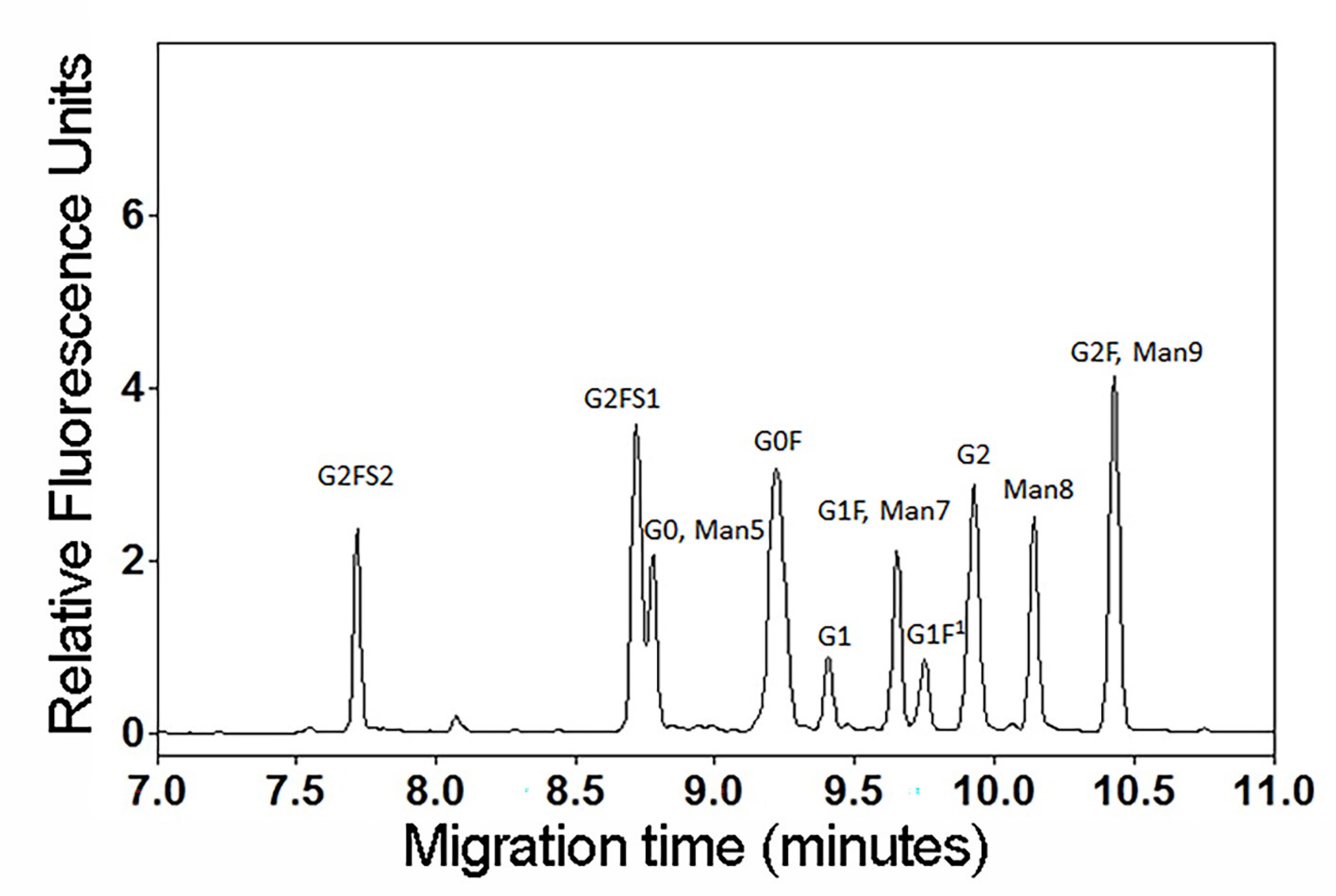

Figure S8. APTS labeled glycan standards run as a mix in CE analysis. Signal detection was by a 488nm laser induced fluorescence (LIF) detector on a PA800 pharmaceutical analysis system (AB Sciex).

Glycan Analysis by HPLC: Profile of Standards (n=3) overlayed

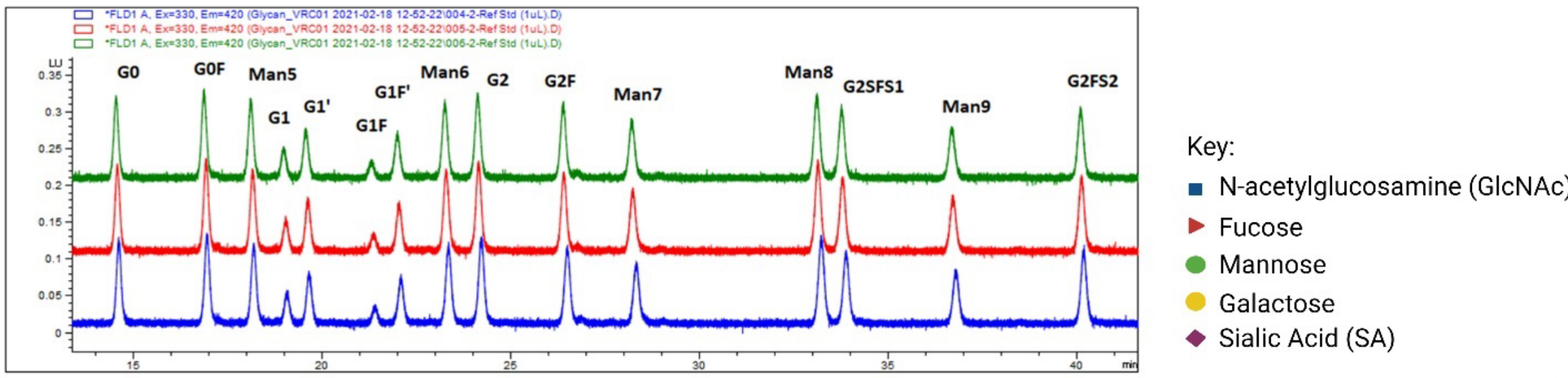

Statistical % RSD of method transfer (n=6)

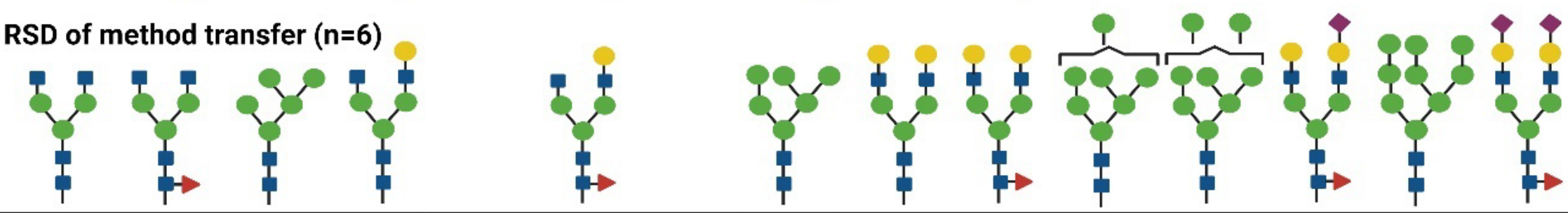

Peak	G0	G0F	Man5	G1	G1′	G1F	G1F′	Man6	G2	G2F	Man7	Man8	G2FS1	Man9	G2FS2
Intraday % RSD	4.58	7.22	2.68	5.60	7.66	7.51	2.49	6.90	9.33	4.69	4.63	2.96	3.46	8.34	5.57
Interday % RSD	7.48	6.75	3.31	5.35	5.00	8.93	6.00	4.02	5.68	1.93	3.43	4.04	4.89	7.28	8.99

Figure S9. Assessment of intraday and inter-day variation in the peak content (area under the curve) for the N-linked 2-AB labeled glycan standards by the HPLC analysis described in the methods section. HPLC quantification involved run time requiring greater than 60 min per sample and some variation in elution times were detected. The extent of variation is presented here to demonstrate the degree of robustness and reproducibility of the assay.

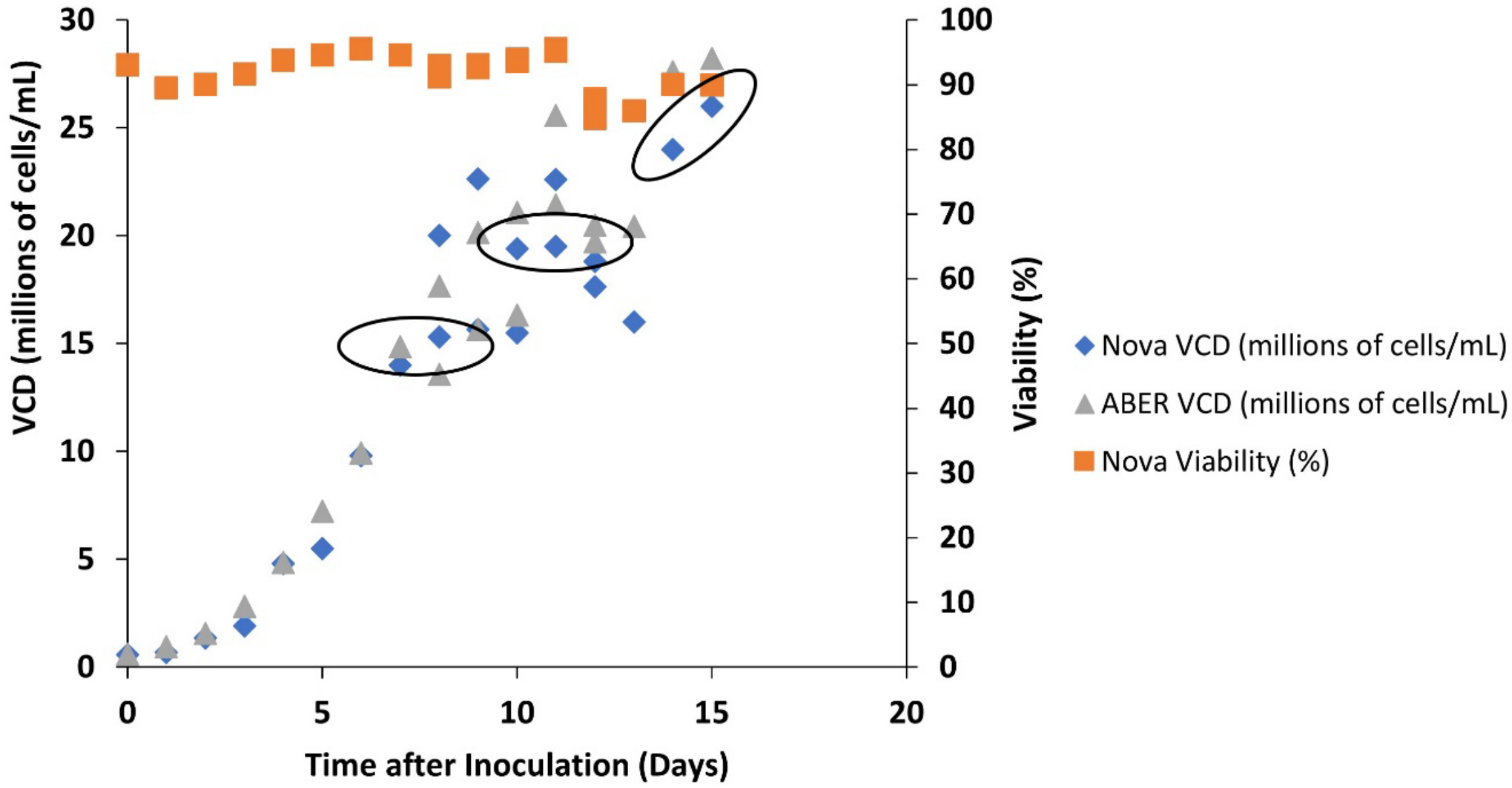

Figure S10 – Viable cell density (VCD) and percent viability of the CHO cells during the bioreactor run. The ellipses on the graph indicate the time points around which the samples were drawn corresponding to the 15-, 20- and 25 × 10E06 cells/mL of CHO cell growth in the bioreactor.

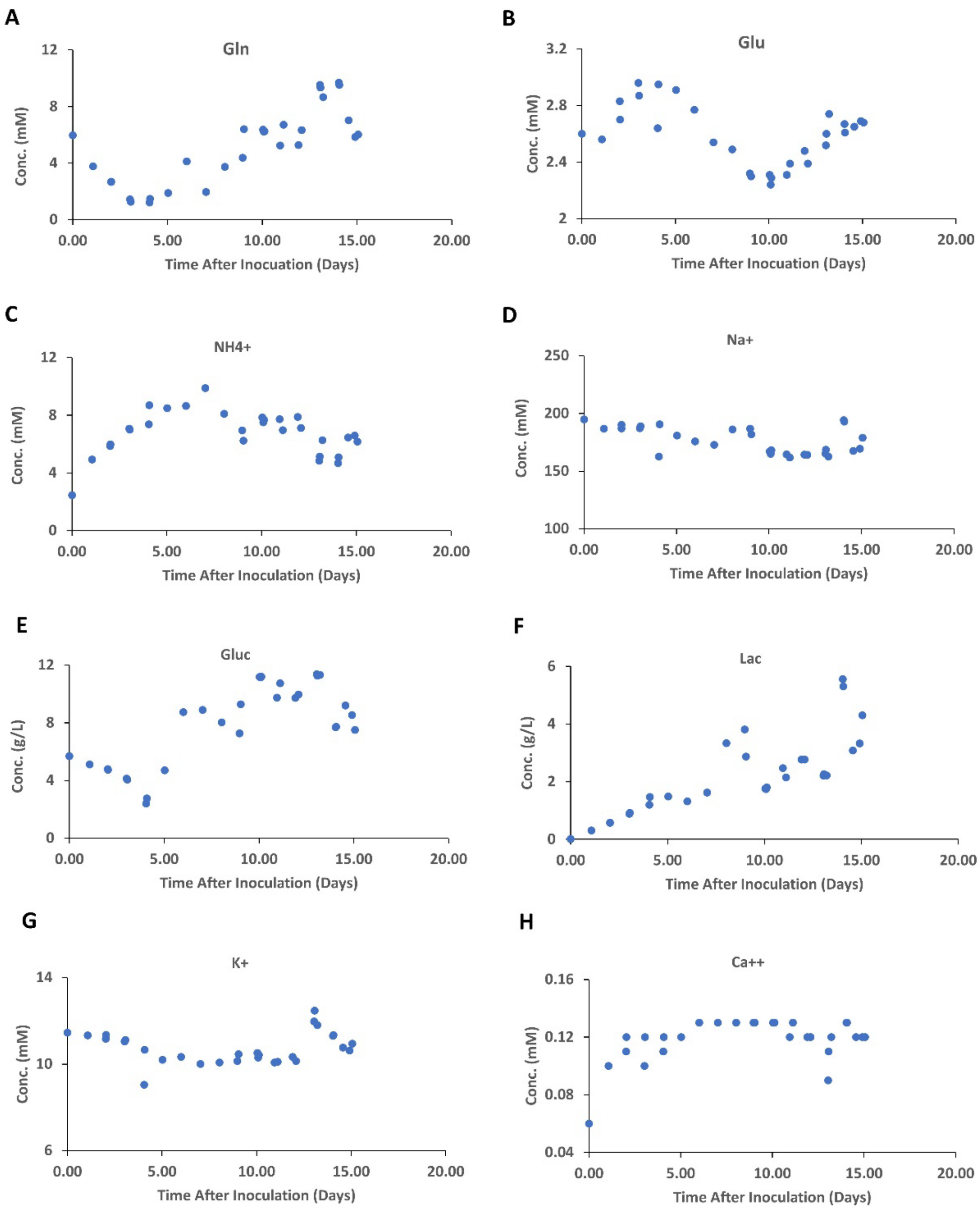

Figure S11 – Metabolite profiles during the bioreactor run as analyzed by Nova Bioprofile Flex2 instrument.

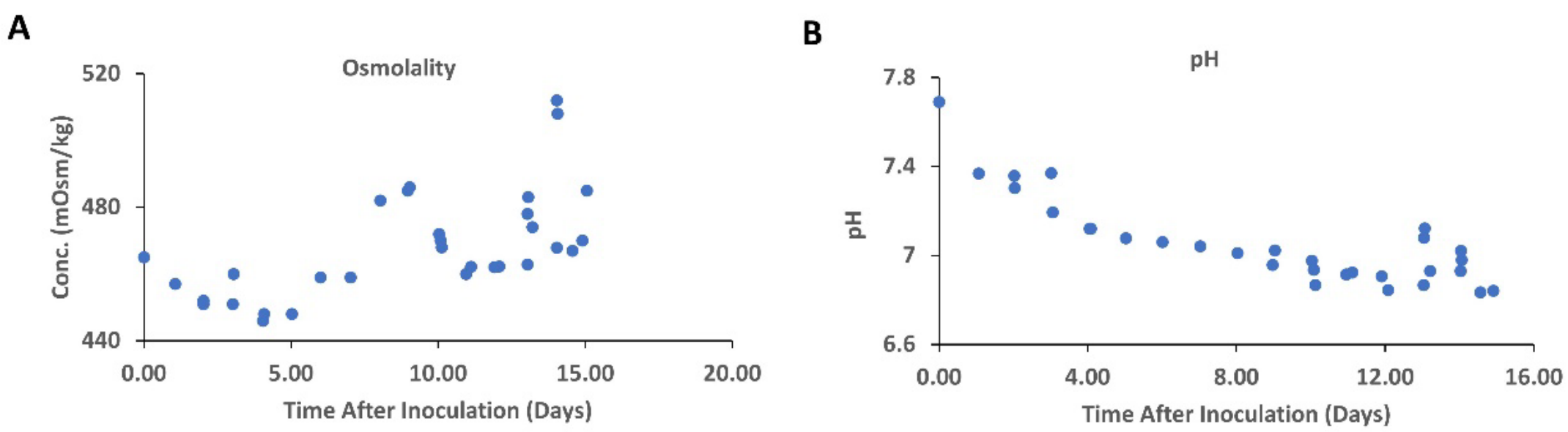

Figure S12 – pH and osmolality (mOsm/kg) of the bioreactor medium through the run as determined on the daily off-line samples using Nova Bioprofile Flex 2 instrument.

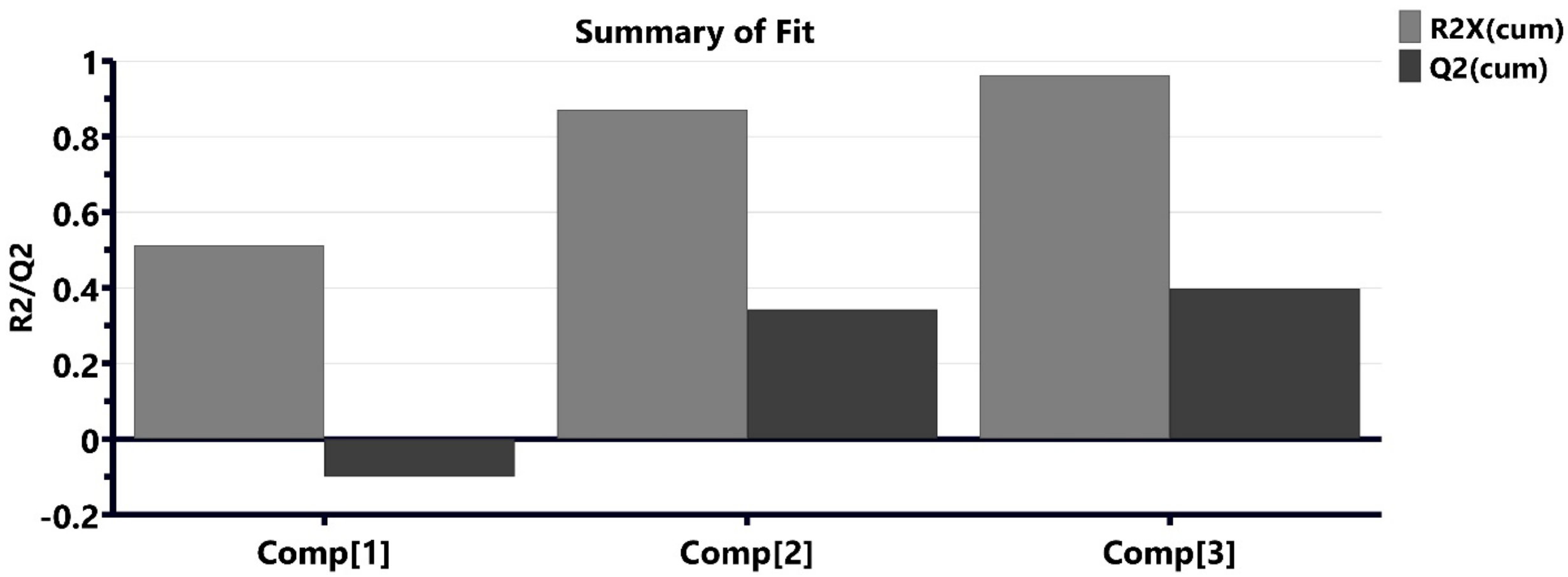

Figure S13. Summary of model fit for the PCA model. This figure indicates how accurate (R2) and predictive (Q2) the model is. The model has a R2 ~ 0.96 and a Q2 ~ 0.39. Three components were utilized for this PCA- X & Y model.

	VCD	Viabillity	Capacitance	Perf. Rate (VVD)	Peak 1	Peak 2	Peak 3	Peak 4	Peak 5	Peak 6 (HHLL)	G2FS 2[a]	G2FS 1[a]	G0, Man 5[a]	G0F[a]	G1F, Man 7[a]	G1F'[a]	G2F[a]	Basic1-pl[b]	Basic2-pl[b]	Basic3-pl[b]	Major-pl[b]	Acidic1-pl[b]	Acidic2-pl[b]	Acidic3-pl[b]	Gluc	Lac	pH
VCD	1	-0.22	0.98	0.79	0.76	-0.9	-0.9	0.73	0.8	-0.77	-0.8	-0.66	0.28	0.97	-0.85	0.31	0.71	0.99	0.22	0.13	-0.94	-0.31	0.022	-0.89	-0.27	0.53	0.42
Viabillity	-0.22	1	-0.18	-0.54	0.36	-0.1	-0.2	0.39	0.29	-0.34	-0.3	-0.49	-1	-0.56	-0.21	-1	-0.91	-0.47	0.84	0.89	0.63	-0.79	0.93	-0.13	0.38	-0.43	0.24
Capacitance	0.98	-0.18	1	0.85	0.8	-0.9	-0.9	0.78	0.84	-0.81	-0.8	-0.71	0.2	0.95	-0.89	0.24	0.65	0.98	0.29	0.2	-0.91	-0.38	0.097	-0.92	-0.13	0.47	0.32
Perf. Rate (VVD)	0.79	-0.54	0.85	1	0.78	-0.9	-0.9	0.76	0.82	-0.79	-0.8	-0.68	0.24	0.96	-0.87	0.28	0.68	0.99	0.25	0.16	-0.93	-0.34	0.056	-0.91	0.018	0.33	-0.15
Peak 1	0.76	0.36	0.8	0.78	1	-1	-1	1	1	-1	-1	-0.99	-0.4	0.57	-0.99	-0.4	0.068	0.66	0.8	0.75	-0.49	-0.86	0.67	-0.97	0.7	-0.2	0.6
Peak 2	-0.89	-0.12	-0.92	-0.91	-1	1	1	-0.96	-1	0.97	0.98	0.93	0.18	-0.75	1	0.15	-0.31	-0.82	-0.63	-0.56	0.69	0.7	-0.47	1	-0.51	-0.05	-0.78
Peak 3	-0.88	-0.15	-0.91	-0.9	-1	1	1	-0.97	-1	0.98	0.98	0.94	0.21	-0.74	1	0.18	-0.28	-0.81	-0.65	-0.58	0.67	0.72	-0.49	1	-0.53	-0.02	-0.76
Peak 4	0.73	0.39	0.78	0.76	1	-1	-1	1	0.99	-1	-1	-0.99	-0.5	0.54	-0.98	-0.4	0.034	0.63	0.82	0.77	-0.46	-0.87	0.7	-0.96	0.73	-0.23	0.58
Peak 5	0.8	0.29	0.84	0.82	1	-1	-1	0.99	1	-1	-1	-0.98	-0.4	0.62	-1	-0.3	0.14	0.71	0.76	0.7	-0.55	-0.82	0.62	-0.99	0.65	-0.13	0.66
Peak 6 (HHLL)	-0.77	-0.34	-0.81	-0.79	-1	0.97	0.98	-1	-1	1	1	0.99	0.41	-0.58	0.99	0.37	-0.08	-0.67	-0.79	-0.74	0.51	0.85	-0.66	0.97	-0.69	0.19	-0.62
G2FS2[a]	-0.78	-0.33	-0.82	-0.8	-1	0.98	0.98	-1	-1	1	1	0.98	0.39	-0.6	0.99	0.36	-0.1	-0.68	-0.78	-0.72	0.52	0.84	-0.65	0.98	-0.68	0.17	-0.63
G2FS1[a]	-0.66	-0.49	-0.71	-0.68	-1	0.93	0.94	-0.99	-1	0.99	0.98	1	0.54	-0.45	0.96	0.51	0.072	-0.55	-0.88	-0.83	0.37	0.92	-0.77	0.93	-0.79	0.33	-0.49
G0, Man5[a]	0.28	-1	0.2	0.24	-0.4	0.18	0.21	-0.45	-0.4	0.41	0.39	0.54	1	0.51	0.27	1	0.88	0.41	-0.88	-0.92	-0.58	0.83	-0.95	0.19	-0.94	0.97	0.47
G0F[a]	0.97	-0.56	0.95	0.96	0.57	-0.8	-0.7	0.54	0.62	-0.58	-0.6	-0.45	0.51	1	-0.69	0.54	0.86	0.99	-0.03	-0.12	-1	-0.063	-0.23	-0.75	-0.19	0.69	1
G1F, Man7[a]	-0.85	-0.21	-0.89	-0.87	-1	1	1	-0.98	-1	0.99	0.99	0.96	0.27	-0.69	1	0.24	-0.22	-0.77	-0.7	-0.64	0.62	0.77	-0.55	1	-0.58	0.046	-0.72
G1F'[a]	0.31	-1	0.24	0.28	-0.4	0.15	0.18	-0.42	-0.3	0.37	0.36	0.51	1	0.54	0.24	1	0.89	0.44	-0.86	-0.9	-0.61	0.81	-0.94	0.16	-0.93	0.98	0.5
G2F[a]	0.71	-0.91	0.65	0.68	0.07	-0.3	-0.3	0.03	0.14	-0.083	-0.1	0.07	0.88	0.86	-0.22	0.89	1	0.8	-0.54	-0.61	-0.9	0.46	-0.69	-0.3	-0.66	0.96	0.84
Basic1-pl[b]	0.99	-0.47	0.98	0.99	0.66	-0.8	-0.8	0.63	0.71	-0.67	-0.7	-0.55	0.41	0.99	-0.77	0.44	0.8	1	0.08	-0.009	-0.98	-0.17	-0.12	-0.82	-0.08	0.61	1
Basic2-pl[b]	0.22	0.84	0.29	0.25	0.8	-0.6	-0.7	0.82	0.76	-0.79	-0.8	-0.88	-0.9	-0.03	-0.7	-0.9	-0.54	0.08	1	1	0.12	-1	0.98	-0.64	0.99	-0.74	0.011
Basic3-pl[b]	0.13	0.89	0.2	0.16	0.75	-0.6	-0.6	0.77	0.7	-0.74	-0.7	-0.83	-0.9	-0.12	-0.64	-0.9	-0.61	-0.009	1	1	0.21	-0.98	0.99	-0.57	1	-0.8	-0.08
Major-pl[b]	-0.94	0.63	-0.91	-0.93	-0.5	0.69	0.67	-0.46	-0.6	0.51	0.52	0.37	-0.6	-1	0.62	-0.6	-0.9	-0.98	0.12	0.21	1	-0.025	0.31	0.69	0.27	-0.75	-0.99
Acidic1-pl[b]	-0.31	-0.79	-0.38	-0.34	-0.9	0.7	0.72	-0.87	-0.8	0.85	0.84	0.92	0.83	-0.06	0.77	0.81	0.46	-0.17	-1	-0.98	-0.03	1	-0.96	0.71	-0.97	0.68	-0.11
Acidic2-pl[b]	0.02	0.93	0.097	0.056	0.67	-0.5	-0.5	0.7	0.62	-0.66	-0.7	-0.77	-1	-0.23	-0.55	-0.9	-0.69	-0.12	0.98	0.99	0.31	-0.96	1	-0.47	1	-0.86	-0.19
Acidic3-pl[b]	-0.89	-0.13	-0.92	-0.91	-1	1	1	-0.96	-1	0.97	0.98	0.93	0.19	-0.75	1	0.16	-0.3	-0.82	-0.64	-0.57	0.69	0.71	-0.47	1	-0.51	-0.04	-0.78
Gluc	-0.27	0.38	-0.13	0.018	0.7	-0.5	-0.5	0.73	0.65	-0.69	-0.7	-0.79	-0.9	-0.19	-0.58	-0.9	-0.66	-0.075	0.99	1	0.27	-0.97	1	-0.51	1	-0.87	-0.4
Lac	0.53	-0.43	0.47	0.33	-0.2	-0	-0	-0.23	-0.1	0.19	0.17	0.33	0.97	0.69	0.05	0.98	0.96	0.61	-0.74	-0.8	-0.75	0.68	-0.86	-0.04	-0.87	1	0.52
pH	0.42	0.24	0.32	-0.15	0.6	-0.8	-0.8	0.58	0.66	-0.62	-0.6	-0.49	0.47	1	-0.72	0.5	0.84	1	0.011	-0.078	-0.99	-0.11	-0.19	-0.78	-0.4	0.52	1

Figure S14. A correlation matrix of different process parameters and measured product quality attributes determined by multivariate data analysis. The cells with thick boundary lines show interesting observations between process parameters and the quality attributes of the mAb.

[a] N-glycan types were analyzed and quantified from whole mAb protein by CE-LIF technique.

[b] The peak area (%) were used for the charge variants determined by cIEF technique
